# Identification of a Nuclear Localization Signal (NLS) in Human Transcription Elongation Factor ELL2

**DOI:** 10.1002/cbf.70019

**Published:** 2024-11-24

**Authors:** Stephan Kohrt, Abarna Baheerathan, Jonas Prokscha, Alexandra Zwosta, Heinrich Sticht, Andrea K. Thoma‐Kress

**Affiliations:** ^1^ Institute of Clinical and Molecular Virology Friedrich‐Alexander‐Universität Erlangen‐Nürnberg (FAU) Erlangen Germany; ^2^ Division of Bioinformatics, Institute of Biochemistry Friedrich‐Alexander‐Universität Erlangen‐Nürnberg (FAU) Erlangen Germany; ^3^ FAU Profile Center Immunomedicine (FAU I‐MED) Friedrich‐Alexander‐Universität Erlangen‐Nürnberg (FAU) Erlangen Germany

**Keywords:** ELL2, NLS, nuclear localization signal, transcription elongation factor

## Abstract

ELL2 is a transcription elongation factor suppressing transcriptional pausing of RNA polymerase II, thereby enhancing gene expression. In accordance with the nuclear localization of ELL2, the protein is supposed to carry out its function in promoting transcription in the nucleus. Yet, it is unknown whether ELL2 carries a nuclear localization signal (NLS). In this study, we identify the NLS of ELL2. In silico analysis resulted in prediction of a strong bipartite NLS with an exceptionally high score at amino acids 311–338 in the conserved region R1 of ELL2. Confocal laser scanning microscopy of a series of ELL2 truncation mutants and quantitative analysis of images verified the presence of R1 to be decisive for nuclear localization of ELL2 suggesting that the predicted NLS is accurate. Deletion of key basic amino acids within the putative NLS in silico and in vitro showed that K319, R320, and K333/K334 are crucial for ELL2's nuclear accumulation, thus confirming the predictions. The isolated ELL2‐NLS was able to translocate an unrelated NLS‐mapping system into the nucleus underlining the strength of the NLS. Taken together, we identified the NLS of ELL2 and mapped individual aa that are crucial for nuclear localization of ELL2.

## Summary

ELL2 is a protein being important for regulation of cellular and viral transcription, a process which takes place in the nucleus. In this study, we identified a sequence stretch within the ELL2 amino acid sequence which is crucial for nuclear localization of ELL2, a so‐called nuclear localization signal (NLS). We used bioinformatics to find the NLS and confirmed its existence by generating several ELL2 mutants that either lacked the NLS or single critical amino acids. By quantitative imaging analysis, we identified four amino acids which are crucial for nuclear localization of ELL2. Finally, we showed that the isolated NLS from ELL2 can also guide other, unrelated proteins into the nucleus, thus, highlighting the strength of the identified NLS.

## 1 Introduction

ELL2, a member of the eleven‐nineteen lysine‐rich leukemia (ELL) family of transcription elongation factors, is highly expressed in pancreas, skeletal muscle, liver, placenta, heart, and in antibody‐secreting plasma cells [1, 2, 3]. Briefly, ELL2 harbors three conserved regions including an N‐terminal R1 region (residues 7–350) responsible for RNA transcription elongation; a central lysine‐rich R2 region (residues 443–474) and a C‐terminal zonula occludens 1 (ZO‐1) binding domain of occludin‐like R3 region (residues 516–640) [1, 4], which is important for ELL2 stabilization and binding to AF4/FMR2 family member 4 (AFF4) or 1 (AFF1) [5, 6].

Functionally, ELL2 is an RNA polymerase II (RNA pol II) elongation factor suppressing transcriptional pausing of RNA pol II and the stoichiometrically limiting component of a so‐called super elongation complex (SEC). The SEC comprises six subunits including one of the scaffold proteins AFF4 or AFF1, which operate as a platform to facilitate the assembly of the SEC. Another important subunit of the SEC is positive transcription elongation factor (P‐TEFb) comprising the catalytic and regulatory subunits cyclin‐dependent kinase 9 (CDK9) and cyclin T1, respectively, which can be released from an inactive complex into active bromodomain‐containing protein 4 (BRD4)/P‐TEFb complexes and the SEC [5, 7, 8, 9, 10, 11]. AFF4 directly binds to the P‐TEFb subunit cyclin T1, and to the other SEC subunits including one of the ELL proteins ELL or ELL2, to eleven‐nineteen leukemia (ENL) or ALL1‐fused gene from chromosome 9 (AF9), and to one of the ELL‐associated factor proteins EAF1 or EAF2 [6, 7, 8, 9, 10, 12, 13]. The SEC is not only required for rapid induction of cellular transcription, for example, in response to stress and for transcription in cancer driven by the protooncogene MYC [9, 14], but also for secretory‐specific immunoglobulin heavy chain production. Specifically, ELL2 contributes to transition of B cells to antibody‐secreting cells by regulating gene expression and splicing patterns in the latter [2, 3, 15]. Cryoelectron microscopy showed that an ELL2‐EAF2 subcomplex directly binds the RNA Pol II lobe domain, which underlines the importance of ELL2 in delivering the SEC to RNA pol II during transcription elongation [16]. A SEC containing ELL2 is also recruited by the viral transactivator protein HIV Tat to induce and increase Tat‐regulated *HIV* proviral transcription at the *HIV* promoter [5, 7, 8, 17]. Of note, ELL2 is upregulated in cells being chronically infected with Human T cell leukemia virus type 1 (HTLV‐1), an oncogenic delta‐retrovirus infecting between 5 and 10 million people worldwide [18, 19]. Further, ELL2 complexes with the viral transactivator and oncoprotein Tax, and it transactivates the *HTLV‐1* promoter [4, 18, 20]. Taken together, the functions of ELL2 on transcription described so far seem to overlap with the localization of ELL2 in the nucleus [1, 7, 18].

The conventional transport of proteins to the nucleus is primarily achieved by the activity of the nuclear localization signal (NLS) of the cargo protein coupled with the two intermediary proteins importin‐α and importin‐β [21, 22, 23, 24]. Distinct binding pockets within importin‐α are able to bind different NLS classes including, amongst others [25, 26], monopartite and bipartite NLS [27]. Monopartite NLS sequences are distinguished by a cluster of basic amino acids (aa), arginines and lysines, whereas bipartite NLS sequences comprise two clusters of basic aa, separated by linker sequences of 10–12 variable aa: (K/R)(K/R)X_10–12_(K/R)_3/5_, where (K/R)_3/5_ indicates that at least three out of five consecutive aa are either lysine or arginine [27, 28, 29, 30, 31]. The NLS sequence is recognized by importin‐α, while importin‐β interacts with constituents of the nuclear pore complex, stimulating the liberation of the cargo protein into the nucleus through the binding of Ran‐GTP [32, 33].

Despite its importance during viral transcription, splicing, and B cell receptor maturation [2, 5, 7, 18], little is known about domains in ELL2 that govern its nuclear localization. Here we report that ELL2 contains an exceptionally strong NLS in its conserved region R1. We provide evidence that basic aa residues K319, R320, and K333/K334 are crucial for nuclear localization of ELL2.

## 2 Materials and Methods

### 2.1 Bioinformatics

Clustal Omega (https://www.ebi.ac.uk/jdispatcher/msa/clustalo; last access on: September 09, 2024) was used to compare the sequence of ELL2 of *Homo sapiens* (GenBank accession no: O00472) with that of *Pan troglodytes* (H2QR91), *Bos taurus* (E1BIK3), *Mus musculus* (Q3UKU1), and *Rattus norvegicus* (A0A8I6A5G5) by multiple sequence alignment. cNLS Mapper (http://nls-mapper.iab.keio.ac.jp/cgi-bin/NLS_Mapper_form.cgi; last access on: September 09, 2024) was used to determine the NLS of ELL2 of the aforementioned species and of the related human ELL (P55199) with a cut‐off score of four and the setting *entire region*, since a score of four or more is assumed to indicate nuclear localization. NLS determination is based on a motif‐scoring algorithm that uses sequence profiles [28]. The Eukaryotic Linear Motif (ELM; http://elm.eu.org; last access on August 27, 2024) resource was used to confirm the bipartite NLS of human ELL2 [34]. To identify phosphorylation sites within the NLS, PhosphoSite Plus (https://www.phosphosite.org/proteinAction.action?id=17483; last access October 15, 2024) was used [35]. AlphaFold 3 (https://alphafoldserver.com/about; last access on: August 28, 2024) was used to predict the structure and interactions between the ELL2‐NLS and other domains within human ELL2 [36]. Structure analysis and visualization was done with RasMol [37].

### 2.2 Cell Culture

HEK293T cells were cultured in Dulbecco's modified eagle medium (Gibco, Life Technologies, Darmstadt, Germany) containing 10% fetal calf serum (anprotec, Bruckberg, Germany), 1% Gluta‐MAX (Gibco, Life Technologies), 0.12 mg/mL penicillin and 0.12 mg/mL streptomycin (both Gibco, Life Technologies).

### 2.3 Plasmids

The following expression plasmids were used: pEF‐1α, empty vector containing a human EF‐1‐α promotor (Life Technologies); pEF‐1α‐ELL2‐myc expressing wildtype ELL2 cloned into pEF‐1α with C‐terminal myc/his‐tag [18]; the ELL2 truncation mutants pEF‐1α‐ELL2‐R1‐myc, pEF‐1α‐ELL2‐N‐myc, pEF‐1α‐ELL2‐∆R2‐myc, pEF‐1α‐ELL2‐R2‐myc, pEF‐1α‐ELL2‐R3‐myc, and pEF‐1α‐ELL2‐C‐myc have been described earlier [4, 38].

### 2.4 Cloning

The following ELL2‐NLS mutants with deletion of the respective single basic aa indicated in brackets were cloned using Gibson assembly: pEF‐1α‐ELL2‐NLS1‐myc (∆R311), pEF‐1α‐ELL2‐NLS2‐myc (∆K319), pEF‐1α‐ELL2‐NLS3‐myc (∆R320), and pEF‐1α‐ELL2‐NLS5‐myc (∆R334). The vector pEF‐1α‐ELL2‐myc [18] served as a template. Two overlapping amplicons were generated for each construct, each ranging from the site to be deleted within the ELL2 NLS up to the *Ampicillin resistance gene* (*amp*) encoded by the vector. Therefore, ELL2‐NLS‐specific forward primers were used together with amp‐specific reverse primers (GA‐amp‐rev) and ELL2‐NLS‐specific reverse primers together with amp‐specific forward primers (GA‐amp‐fwd; Supporting Table 1), respectively. Briefly, the two amplicons were purified and linked using Gibson assembly enzyme mix (Gibson Assembly Master Mix, New England BioLabs, Ipswich, MA, USA) at 50°C for 1 h and transformed into chemically competent XL1‐blue cells according to the manufacturer's protocol. Plasmids pEF‐1α‐ELL2‐NLS6‐myc (∆R336) and pEF‐1α‐ELL2‐∆NLS‐myc (∆R320‐S338) were cloned by ShineGene Bio‐Technologies, Inc (Shanghai, China).

The NLS‐mapping vector pHM830 contains an N‐terminal GFP‐expression cassette followed by a multiple cloning site and a C‐terminal β‐galactosidase (ß‐Gal) expression cassette. The vector was kindly provided by T. Stamminger (Ulm, Germany) and M. Marschall (Erlangen, Germany). To generate GFP‐ELL2‐β‐Gal NLS construct for testing the isolated ELL2 NLS at aa 311–338 and deletions thereof lacking individual basic aa (ΔR311, ΔK319, ΔR320, and ΔK334), extended oligonucleotides were designed to contain *Afl*II and *Xba*I restriction sites flanking the ELL2 NLS sequence or the desired NLS deletion mutants. The oligonucleotides were inserted into pHM830 by standard cloning procedures. Oligos are listed in Supporting Information S1: Table 1. The integrity of coding sequences of all plasmids was verified by automated sequencing (Macrogen, Amsterdam, Netherlands) and analyzed using Vector NTI Advanced ContigExpress 9.1 (Life Technologies).

### 2.5 Transfections

For immunofluorescence analysis 5 × 10^5^ 293T HEK cells, and for western blot analysis, 2.5 × 10^5^ 293T HEK cells were seeded on cover slips in six well plates or directly into six well plates, respectively, and transfected with a total amount of 2 µg or 1 µg of corresponding plasmids, respectively, using GeneJuice transfection reagent (Merck Millipore, Darmstadt, Germany) according to the manufacturer's protocol 24 h later. Cells were analyzed 48 h after transfection.

### 2.6 Confocal Laser Scanning Microscopy

At 48 h of posttransfection, cells were washed twice with PBSo (phosphate‐buffered saline without Ca^2+^ and Mg^2+^) and fixed with 2% para‐formaldehyde (PFA, 1 h, 20°C). After washing twice with PBSo/1% fetal calf serum (FCS)/0.3% saponin, cells were blocked in PBSo containing 5% FCS for 1 h at 20°C. Afterwards, cells were washed once with PBSo/1% FCS/0.3% saponin and stained with primary antibodies mouse anti‐myc (1:1000, ab32, Abcam, Cambridge, UK) in PBSo/1% FCS/0.5% Saponin for 1 h at 37°C. After two wash steps with PBSo/1% FCS/0.3% saponin, cells were stained with secondary antibodies anti‐mouse AlexaFluor 488 (Life Technologies), in PBSo/1% FCS/0.5% saponin for 30 min at 37°C, followed by two washing steps with PBSo/1% FCS/0.3% saponin and one washing step with PBSo. The nuclei were stained with ProLong Gold antifade reagent with DAPI (Life Technologies) and analyzed on a confocal laser scanning microscopy Leica TCS SP5 facilitated with a 63 × 1.4 HCX PL AP0 CS oil immersion objective lens. Images were analyzed with LAS AF Lite software (Leica, Wetzlar, Germany), and the frequency of cells with nuclear and/or cytoplasmic localization of proteins in 3–15 optical fields were quantitatively evaluated as specified in the respective figure legends. The mean fluorescence intensities (MFIs) of myc‐specific fluorescence or of GFP in 10 individual cells per construct were analyzed as described previously [39]. Briefly, a nuclear and whole‐cell region of interest (ROI) was defined, and the respective fluorescence intensities were analyzed using Leica LAF AF Lite software. The cytoplasmic ROI was calculated by subtracting the nuclear ROI from the whole‐cell ROI. The MFI was calculated for the nuclear (Fn) and the cytoplasmic (Fc) fluorescence by dividing the pixel sum by the pixel count. A background MFI was recorded for each image and subtracted from the nuclear and cytoplasmic MFIs. Then, the ratio between the nuclear and cytoplasmic MFIs (*Fn/c*) was calculated for each cell. Mean *Fn/c* values ± SEM are displayed.

### 2.7 Western Blot

At 48 h posttransfection, cells were washed in PBS (without Ca^2+^ and Mg^2+^) and spun down (7000 rpm, 4°C, 7 min). Pellets were lysed in TNE lysis buffer (10 M NaCl, 10 mM TRis/HCl (pH 7.0), 10 mM EDTA, 1% Triton X‐100, 2 mM DTT and protease inhibitors (20 µg/mL leupeptin, 20 µg/mL aprotinin and 1 mM phenylmethylsulfonyl fluoride). Afterward, lysates were sonicated five times for 30 s (Bransons Ultrasonics Analog Sonfier Modell 450, output control = 8, duty cycle = 60, 5 × 30 s, 4°C) and spun down at 4°C. Protein concentrations of the cleared supernatants were adjusted to equal amounts (50–70 µg) and samples were boiled in sodium dodecyl sulfate (SDS) loading dye (10 mM Tris/HCl (pH 6.8), 10% glycerol, 2% SDS, 0.1% bromophenyl blue, 5% β‐mercaptoethanol) for 10 min at 95°C. Separation of the proteins was performed via SDS‐PAGE on 12% polyacrylamide gels. PageRuler Prestained Protein Ladder (Thermo Scientific) served as molecular weight marker. Transfer of the separated proteins onto the nitrocellulose membrane (Whatmann, Protran, Whatmann GmbH, Dassel, Germany) was conducted by using standard techniques. The membrane was incubated in 1X T‐TBS/0.1% Tween‐20/5% FCS for 1 h at 20°C to block unspecific binding followed by incubation with primary mouse monoclonal antibodies anti‐Myc (9B11, Cell Signaling Technologies, Danvers, MA, USA), anti‐GFP (9F9.F9, Abcam) and mouse anti‐GAPDH (sc‐47724, Santa Cruz Biotechnology, Dallas, TX, USA). As secondary antibodies, Horseradish peroxidase‐coupled anti‐mouse secondary antibodies from GE Healthcare (GE Healthcare, Little Chalfont, UK) were used. The enhanced chemiluminescence activity induced by the horseradish peroxidase was detected with a CCD camera (ChemoStar, Intas Science Imaging GmbH, Göttingen, Germany).

### 2.8 Statistics

Microsoft Office Excel (Microsoft Corporation, Redmond, WA, USA) and GraphPad PRISM version 9.5.1 (GraphPad Software, Boston, MA, USA) were utilized to determine significant differences between numerical results. The normal distribution of the measured values was checked using the Shapiro–Wilk test, and nonnormally distributed data were compared using Kruskal–Wallis test and Dunn's multiple comparisons test. *p*‐values of < 0.05 (*), < 0.01 (**), < 0.001 (***), or < 0.0001 (****) were considered to be significant.

## 3 Results

### 3.1 Computational Analysis Predicts a Strong Bipartite NLS in ELL2 R1, Which Is Crucial for Nuclear Localization of ELL2

**Figure 1:**
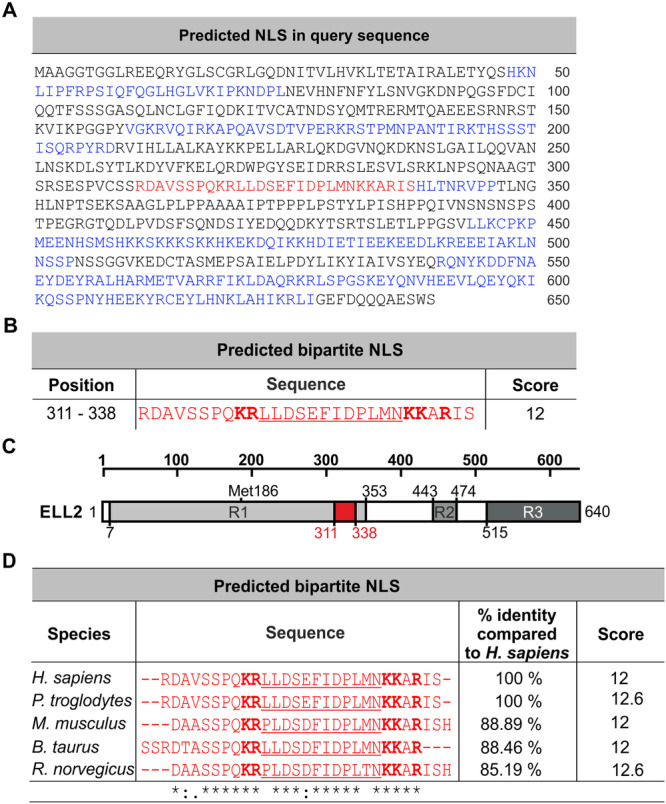
Bioinformatics predicts a strong bipartite nuclear localization signal (NLS) in ELL2. (A) Amino acid (aa) sequence of ELL2 and results from cNLS Mapper with low‐scoring NLS sequences (blue) and an exceptional high‐scoring NLS sequence (red). (B) Depiction of the bipartite NLS with the highest score. Bold: motif‐specific basic aa in NLS; underlined: linker. (C) Scheme of ELL2 with localization of the putative NLS sequence highlighted in red. R1, R2, and R3, conserved regions 1, 2, and 3, respectively. Met 186, alternative start codon at Met 186. (D) Multiple sequence alignment of ELL2 NLS sequences of different species and the percent of identity matrix (PIM) compared to human ELL2. Score indicates results from cNLS Mapper . (dot) indicates a conserved position with similar but not identical residues; * (asterisk), a fully conserved position where all the aligned residues are identical; : (colon), a conserved position where the aligned residues are strongly similar in their biochemical properties (e.g., hydrophobicity, charge, and size).

**Figure 2:**
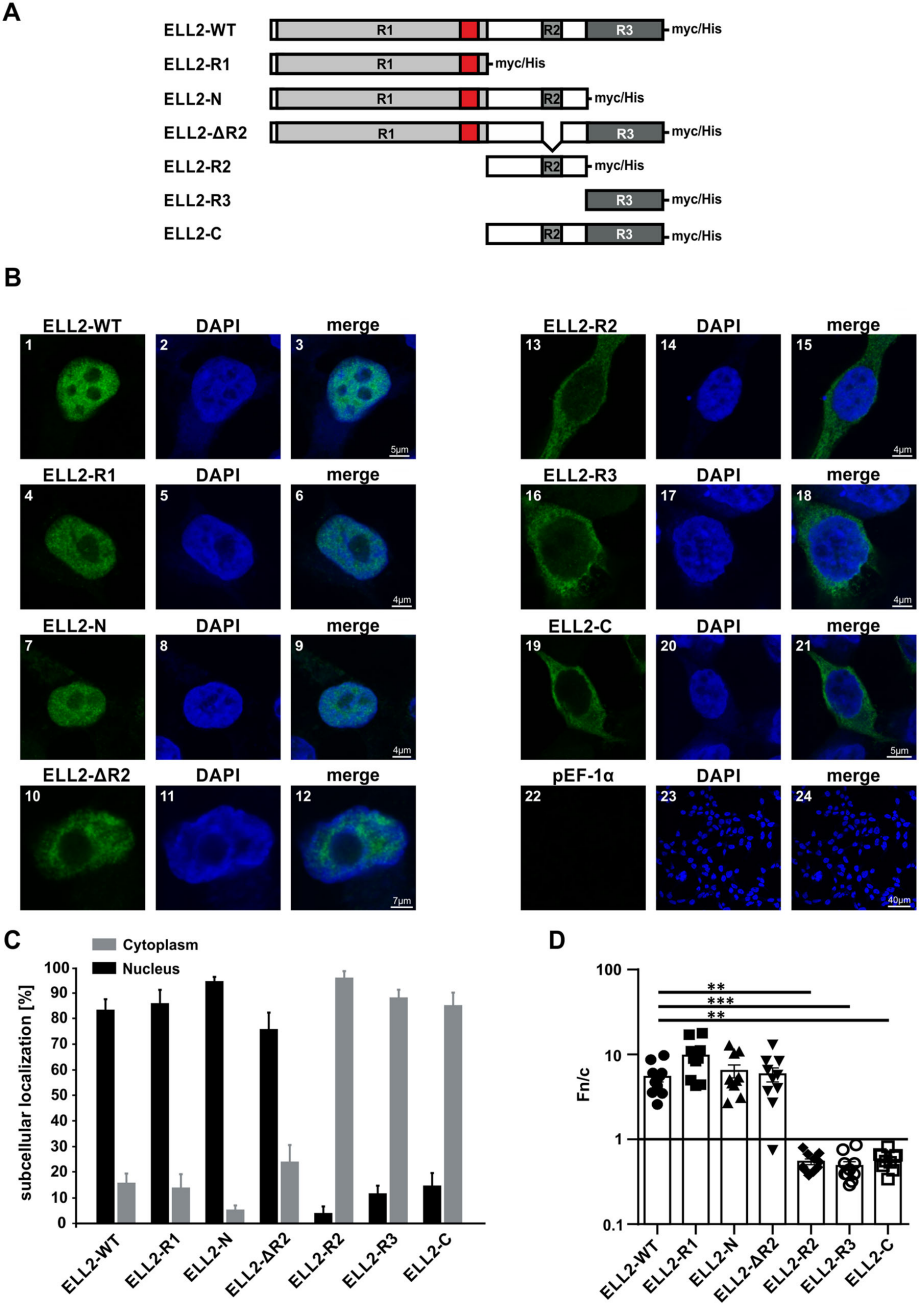
The ELL2 region R1 is crucial for nuclear localization of ELL2. (A) Schematic representation of ELL2 wildtype (ELL2‐WT) and ELL2 truncation mutants with C‐terminal myc‐his tag. R1, R2, and R3, conserved regions 1, 2, and 3, respectively. Red box: localization of putative NLS. (B) Immunofluorescence analysis of ELL2‐WT, ELL2 truncations, and the nucleus was conducted in 293T cells. Cells were transfected with 1 µg of expression plasmids pEF‐ELL2‐myc (ELL2‐WT, 1–3), pEF‐ELL2‐R1‐myc (ELL2‐R1, 4–6), pEF‐ELL2‐N‐myc (ELL2‐N, 7–9), pEF‐ELL2‐ΔR2‐myc (ELL2‐ΔR2, 10–12), pEF‐ELL2‐R2‐myc (ELL2‐R2, 13–14), pEF‐ELL2‐R3‐myc (ELL2‐R3, 16–18), pEF‐ELL2‐C‐myc (ELL2‐C, 19–21) or an empty vector control (pEF1α, 22–24). After 48 h, cells were stained intracellularly with primary mouse anti‐myc followed by anti‐mouse Alexa Fluor 488 (green) antibodies. Nuclei were counterstained with DAPI (blue). Images were acquired on a Leica TCS SP5 confocal laser scanning microscope with a 63 × 1.4 HCX PL APO CS oil immersion objective. Images depicting ELL2‐WT and ELL2 truncations (green), the nucleus (blue), and the merged stains are displayed. Scale bars indicate 4, 5, 7, or 40 µm as indicated. (C) Quantitation of images as displayed in B and Supporting Information S1: Figure S2. At least 182 cells in 15 optical fields of four independent experiments were analyzed. The frequency of cells expressing ELL2‐WT or ELL2 truncation mutants in the nucleus (black) or the cytoplasm (gray) is indicated. (D) The ratio of mean fluorescence intensities between proteins located in the nucleus and the cytoplasm (*Fn/c*) in single cells like those shown in (B) expressing the indicated ELL2 constructs are shown. Each data point corresponds to quantification of a single cell, bars indicate mean values. Ten randomly selected cells per condition were analyzed, and values were compared to those obtained upon expression of ELL2‐WT using Kruskal–Wallis test and Dunn's multiple comparisons test (**p < 0.01; ***p < 0.001).

To identify the NLS in ELL2, cNLS Mapper was applied to predict the likelihood of the aa sequence of ELL2 to contain an NLS by using a scoring system [28]. cNLS Mapper identified 18 putative NLS‐like sequence stretches (Figure 1A, blue and red, Supporting Information S1: Figure S1), one exhibiting an exceptional high score of 12 at aa 311–338 of ELL2 (Figure 1B, red; Supporting Information S1: Figure S1A). Interestingly, an NLS with the same high score of 12 was identified in the related ELL at a similar position (aa 316–342 of ELL; Supporting Information S1: Figure S1B). Briefly, cNLS Mapper assumes that an NLS with a score ranging between 8 and 10 indicates sole nuclear localizaton of the corresponding protein. With a score of three to five, localization in both the nucleus and cytoplasm is possible, a score between six and eight indicates a partial nuclear localization, while a score of one to two suggests the protein is primarily located in the cytoplasm [28]. As a score greater than 10 strongly suggests nuclear localization, the anticipated NLS sequence of ELL2 with the high score of 12 at aa 311–338 was selected for further analysis. The predicted ELL2 NLS is classified as a bipartite NLS [27, 30], as denoted by two basic aa stretches (Figure 1B, bold) connected through a linker sequence (Figure 1B, underlined) and localizes within the R1 region of ELL2 (Figure 1C). Such sequences are capable of binding to two distinct binding pockets within importin‐α and thus can be transported into the nucleus via interaction with importin‐β1, as indicated by previous studies [24, 27, 28, 32, 33]. The bipartite NLS in ELL2 could also be identified with ELM [34], another predicting tool (Supporting Information S1: Figure S1C). Moreover, we found a high percent identity and a high score of the NLS in ELL2 sequences of other species than *H. sapiens* including *P. troglodytes*, *M. musculus*, *B. taurus*, and *R. norvegicus* (Figure 1D), thus, suggesting evolutionary conservation of the NLS. Since the putative NLS with the highest score was identified to localize within the conserved region R1 of human ELL2, which also plays a significant role in enhancing Tax‐mediated viral transactivation and transcription elongation [1, 4, 18], the subcellular localization of a series of human ELL2 truncation mutants was assessed. For this purpose, myc‐tagged ELL2 truncation mutants encoding R1 and the putative NLS (Figure 2A; ELL2‐R1, ELL2‐N, ELL2∆R2) or lacking R1 and the NLS (Figure 2A; ELL2‐R2, ELL2‐R3, ELL2‐C) were compared with ELL2 wildtype (ELL2) upon transfection into 293 T cells and analysis by confocal laser scanning microscopy. The presence of all ELL2 constructs was detected using anti‐myc antibodies. Representative single‐cell images (Figure 2B) and overview images (Supporting Information S1: Figure S2) of all ELL2 truncation mutants are shown. As anticipated, ELL2 was predominantly located in the nucleus, excluding the nucleoli (Figure 2B, 1–3), thus, confirming earlier observations [4]. A localization pattern comparable to ELL2‐WT (Figure 2B, 1–3) could be observed with the mutants ELL2‐R1, ELL2‐N, and ELL2‐ΔR2 (Figures 2B, 4–12), all containing R1 and the putative NLS. Contrary, ELL2‐R2, ELL2‐R3, and ELL2‐C (Figure 2B, 13–21), all lacking R1, were predominantly found in the cytoplasm. These observations are backed by quantifying the frequency of cells expressing the respective proteins in the nucleus or the cytoplasm in a series of overview images of each ELL2 truncation mutant, underlining the findings with quantitative data (Figure 2C). Moreover, a more detailed analysis quantitating the nuclear accumulation of ELL2 proteins at the single cell level by calculating the ratio between the nuclear and cytoplasmic mean flurescence intensities (*Fn/c*) confirmed our findings (Figure 2D). As expected, ELL2‐WT accumulated in the nucleus with a *Fn/c* of 5.5 ± 0.7. Nuclear accumulation was also found for ELL2‐R1 (*Fn/c* = 9.695 ± 1.5), ELL2‐N (*Fn/c* = 6.4 ± 1.1) and ELL2‐∆R2 (*Fn/c* = 5.8 ± 1.1) and did not differ significantly compared to ELL2‐WT. On the other hand, ELL2‐R2, ELL2‐R3, and ELL2‐C were mainly accumulating in the cytoplasm with mean *Fn/c* values below 0.6 and differed significantly from ELL2‐WT (*p* < 0.01). Together, our data support the initial predictions that ELL2 contains an NLS within R1, which may explain the primarily nuclear localization of ELL2 [1, 4, 18].

**Figure 3:**
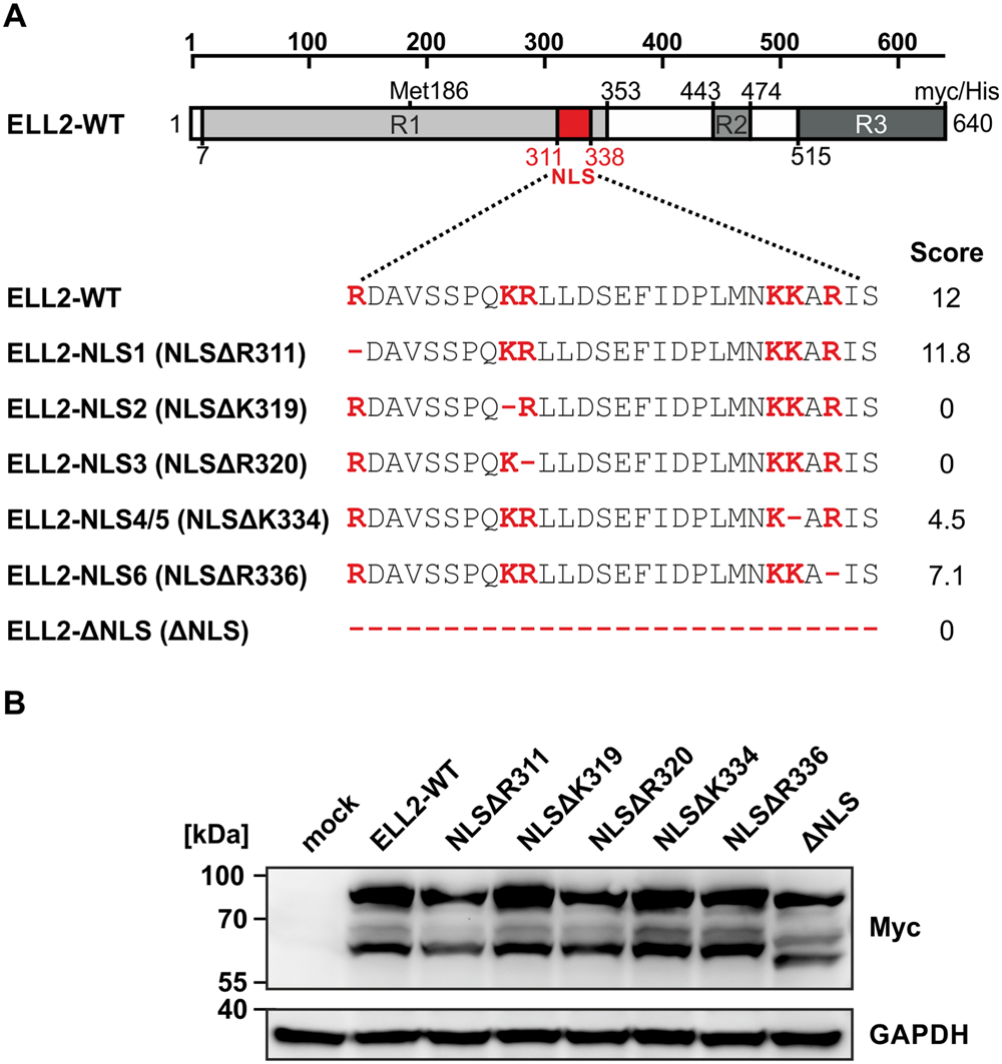
In silico and in vitro mutagenesis of the ELL2 NLS. (A) Schematic overview of ELL2‐WT and ELL2 deletion mutants. The ELL2‐WT aa sequence carrying the predicted nuclear localization signal (NLS, aa 311–338), in silico designed deletion mutants (minus, red) of all basic amino acids (bold, red) within the predicted ELL2 NLS (ELL2‐NLS1 to ELL2‐NLS6), or deletions of the whole NLS (ELL2‐∆NLS) were analyzed by cNLS Mapper. Scores of the respective sequences are indicated. (B) Test expression of ELL2‐NLS deletion mutants upon transfection of 293T cells with 1 µg of myc‐tagged expression plasmids, including pEF‐1α‐ELL2‐myc (ELL2), ELL2 truncations pEF‐1α‐ELL2‐NLS‐1‐myc (NLSΔR311), pEF‐1α‐ELL2‐NLS‐2‐myc (NL2ΔK319), pEF‐1α‐ELL2‐NLS‐3‐myc (NLSΔR320), pEF‐1α‐ELL2‐NLS‐5‐myc (NLSΔK334), and the control vector pEF‐1α (mock). After 48 h, Western Blot analysis was performed using antibodies specific for myc and the housekeeping gene glyceraldehyde 3‐phosphate dehydrogenase (GAPDH). Images were cropped due to technical reasons and original blots are presented in Supporting Information S1: Figure S4.

**Figure 4:**
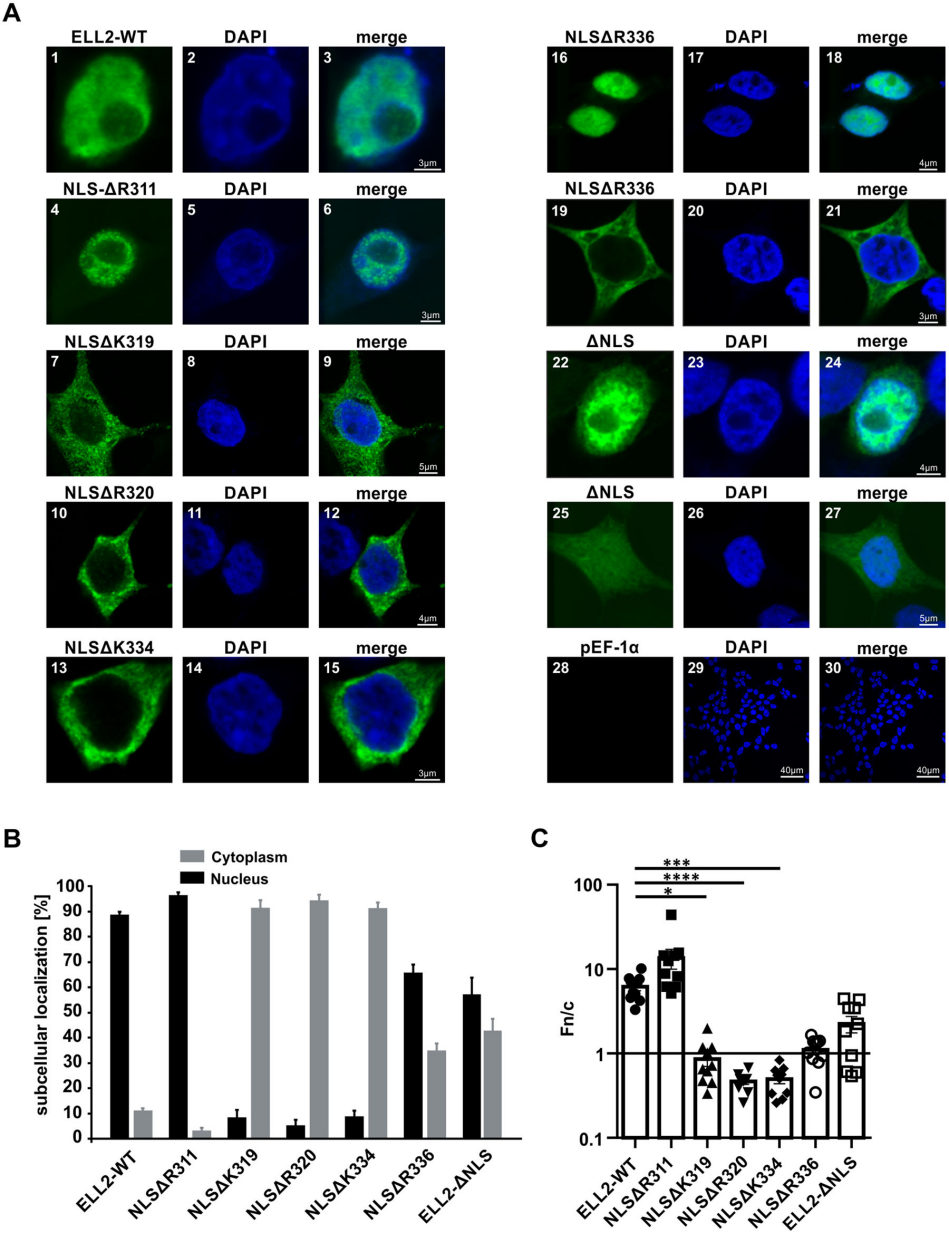
Basic residues K319, R320, K333/K334 are essential for the nuclear localization of ELL2. (A) Immunofluorescence analysis of ELL2‐WT, ELL2‐NLS mutants and the nucleus in 293T cells transfected with the expression plasmids (1 µg each) pEF‐1α‐ELL2‐myc (ELL2‐WT, 1–3), ELL2 deletion mutants pEF‐1α‐ELL2‐NLS‐1‐myc (NLSΔR311, 4–6), pEF‐1α‐ELL2‐NLS‐2‐myc (NL2ΔK319, 7–9), pEF‐1α‐ELL2‐NLS‐3‐myc (NLSΔR320, 10–12), pEF‐1α‐ELL2‐NLS‐4/5‐myc (NLSΔK334, 13–15), pEF‐1α‐ELL2‐NLS‐6‐myc (NLSΔR336, 16–21), pEF‐1α‐ELL2‐∆NLS‐myc (∆NLS, 22–27), or the empty vector pEF‐1α (28–30) as negative control. After 48 h, cells were stained intracellularly with primary mouse anti‐myc followed by anti‐mouse Alexa Fluor 488 (green) antibodies. Nuclei were counterstained with DAPI (blue). Images were acquired on a Leica TCS SP5 confocal laser scanning microscope with a 63 × 1.4 HCX PL APO CS oil immersion objective. Images depicting ELL2‐WT and ELL2‐NLS deletion mutants (green), the nucleus (blue), and the merged stains are displayed. Scale bars indicate 3, 4, 5, or 40 µm as indicated. (B) Quantitative analysis of images as displayed in B and Supporting Information S1: Figure S5. At least 64 cells in three optical fields of three independent experiments were analyzed. The frequency of cells expressing ELL2‐WT or ELL2‐NLS deletion mutants in the nucleus (black) or the cytoplasm (gray) is indicated. (C) The ratio of mean fluorescence intensities between proteins located in the nucleus and the cytoplasm (*Fn/c*) in single cells like those shown in (B) expressing the indicated ELL2 constructs are shown. Each data point corresponds to quantification of a single cell, bars indicate mean values. Ten randomly selected cells per condition were analyzed, and values were compared to those obtained upon expression of ELL2‐WT using Kruskal–Wallis test and Dunn's multiple comparisons test (*p < 0.05; ***p < 0.001; ****p < 0.0001).

### 3.2 In silico Mutagenesis of the ELL2 NLS Identifies Potential Important Residues for Nuclear Localization of ELL2

Generally, the composition of two clusters of basic aa separated by an intervening sequence stretch of about 10–12 aa is decisive for the formation of a bipartite NLS ((K/R)(K/R)X_10–12_(K/R)_3/5_) [24, 27, 30]. To identify more precisely which basic aa are important for nuclear localization of ELL2, we systematically deleted all basic aa R and K residues in silico and re‐analyzed the resulting ELL2 sequence using cNLS Mapper (Figure 3A). Deletion of the initial R (R311) displayed negligible effects on the score (score 11.8) being in line with the fact that R311 is not part of the (K/R)(K/R)X_10–12_(K/R)_3/5_ consensus sequence (Figure 1B). Contrary, deletion of K319 or R320 resembling the two basic aa upstream of the linker region within the NLS completely destroyed the predicted NLS as indicated by the score of 0, thus, achieving the same results as upon complete deletion of the NLS (ΔNLS) (Figure 3A). According to cNLS Mapper, the resulting score of 4.5 for NLSΔK333 or NLSΔK334 did not predict explicit subcellular localization, as both nuclear and cytoplasmic localization are possible [28]. Finally, a NLS could still be determined upon deletion of R336 (NLSΔ336), although its score of 7.1 is much lower than that of ELL2‐WT. Together, in silico analysis suggests that deletions of core aa upstream of the linker region (K319, R320) impair the NLS to a much greater extent than deletions of aa following the linker region (K333, K334, R336). In silico substitution of all basic aa by alanine gave similar trends (Supporting Information S1: Figure S3; Figure 3A). Since cNLS Mapper only provides predictions, it is important to consider the limitations of the in silico analysis. To confirm the possible NLS in vitro, we generated ELL2‐NLS deletion mutants (Figure 3B; Supporting Information S1: Figure S4). Western Blot analysis of the mutants revealed that all constructs were properly expressed and exhibited the typical ELL2‐specific expression pattern: the band at around 70 kDa corresponds to unmodified ELL2 whilst the band at around 90 kDa corresponds to posttranslational modifications of ELL2. Moreover, a slower‐migrating band at about 60 kDa indicates that the ELL2 has been translated using an alternative start codon (M186) or processed via a cleavage site near Met186 [2, 4, 5].

### 3.3 Basic AA Residues K319, R320, K333/334 of the ELL2‐NLS Are Crucial for Nuclear Localization of Wildtype ELL2

To confirm the effects suggested by the in silico predictions, the influence of the different basic aa on the resulting subcellular localization of ELL2 was investigated using the generated ELL2‐NLS deletion mutants and confocal laser scanning microscopy. Therefore, 293 T cells were transfected with the respective constructs and after 48 h, cells expressing ELL2 or ELL2‐deletion mutants (Figure 4A, 1–27; Supporting Information S1: Figures S5, 1–21) or mock‐transfected control cells (Figure 4A, 28–30; Supporting Information S1: Figure S5, 22–24) were stained with anti‐myc and Alexa Fluor 488‐coupled secondary antibodies. For better discrimination of ELL2's subcellular localization, nuclei were stained using DAPI. For ELL2‐WT, nuclear localization could be confirmed for both single cell (Figure 4A, 1–3) and overview images (Supporting Information S1: Figure S5, 1–3) as shown previously (Figure 2, 1–3). Moreover, the frequency of cells expressing the respective proteins predominantly in the nucleus or the cytoplasm was evaluated (Figure 4B) resulting in nearly 89% of cells expressing ELL2 in the nucleus. Expression of NLSΔR311 (score 11.8; Figure 4A, 4–6; Supporting Information S1: Figure S5, 4–6) was detectable in the nucleus and comparable to ELL2‐WT (ca. 97% nuclear localization). Confirming the in silico predictions (Figure 3A), both mutants NLSΔK319 (Figure 4A, 7–9; Supporting Information S1: Figure S5, 7–9) and NLSΔR320 (Figure 4A, 10–12; Supporting Information S1: Figure S5, 10–12), each with a predicted score of 0, showed predominant localization in the cytoplasm (91% or 95% cytoplasmatic localization, respectively; Figure 4B). Interestingly, NLSΔK334 (Figure 4A, 13–15; Supporting Information S1: Figure S5, 13–15) with a score of 4.5 was not able to localize in the nucleus, as a predominant cytoplasmatic localization could be observed here as well (91% cytoplasmatic localization, Figure 4B). We did not perform a separate analysis of NLSΔK333, because deletion of K333 results in the same overall aa sequence as deletion of the adjacent K334. Analysis of NLSΔR336 (score 7.1, Figure 3A) revealed a mixed phenotype since both (Supporting Information S1: Figure S5, 16–18), nuclear (Figure 4A, 16–18; 66% of cells, Figure 4B) or cytoplasmatic (Figure 4A, 19–21; 34% of cells, Figure 4B) localization was detectable. Surprisingly, ELL2ΔNLS exhibited a similar phenotype showing both (Supporting Information S1: Figure S5, 19–21), either nuclear (Figure 4A, 22–24; 59% of cells, Figure 4B) or cytoplasmatic (Figure 4A, 25–27; 41% of cells, Figure 4B) localization. To shed more light on this issue, quantitative analysis of ELL2's nuclear accumulation was performed at the single cell level, which confirmed our findings that ELL2‐WT and NLSΔR311 accumulate in the nucleus with a *Fn/c* of 6.2 ± 0.7 and 13.55 ± 3.6, respectively (Figure 4C). The localization of NLSΔK319, NLSΔR320 and NLSΔK334 differed significantly from ELL2‐WT (*p* < 0.05, *p* < 0.0001, *p* < 0.001, respectively), and these mutants exhibited *Fn/c* values below 1 indicating predominant cytoplasmic localization. NLSΔR336 (*Fn/c* = 1.1 ± 0.1) was almost evenly distributed between the nucleus and the cytoplasm (Figure 4C), thus, reflecting the initially observed phenotype (Figure 4A, 16–18, 19–21; Figure 4B). Interestingly, quantitation of ELL2ΔNLS' nuclear accumulation revealed that despite deletion of the NLS, this mutant exhibited impaired nuclear accumulation compared to ELL2‐WT (*Fn/c* = 2.3 ± 0.5 vs. F/nc = 6.2 ± 0.7), but the difference was not statistically significant (Figure 4C). Since ELL2ΔNLS lacks a larger sequence stretch (aa 311–338) compared to the single aa deletions, this may cause a more drastic change of ELL2 folding, thus resulting in secondary effects impairing ELL2's nuclear localization. To address this aspect in more detail, we modeled the structure of full‐length ELL2 including potential posttranslational modifications in the region of the NLS. A phosphosite analysis revealed that S316 located in the NLS of human ELL2 is phosphorylated (https://www.phosphosite.org/proteinAction.action?id=17483). Interestingly, also the NLS of the highly homologous mouse ortholog (Figure 1D) is phosphorylated at S314, S315 (corresponds to S316 of human ELL2), and S323, suggesting conserved functions. Modeling of the ELL2 structure with AlphaFold3 suggests that the central spacer of the NLS (residues 324–330) interacts with the R3 domain (Supporting Information S1: Figure S6). Interestingly, this interaction masks residues 541–556 of R3, which are part of a weak bipartite NLS spanning residues 541–570 (Supporting Information S1: Figure S1A). Upon deletion of residues 311–338 this weak NLS would become accessible, thus providing a potential explanation for the localization of ELL2‐Δ‐NLS in both the nucleus and the cytoplasm (Figure 4). Together, our findings indicate that R311 is not required for full nuclear translocation of ELL2 while R336 is partially relevant. However, single basic aa K319, R320, or K333/K334 within the newly identified NLS in R1 are crucial for nuclear localization of ELL2.

### 3.4 The ELL2 NLS Sequence Can be Confirmed With an Established NLS Mapping System

**Figure 5:**
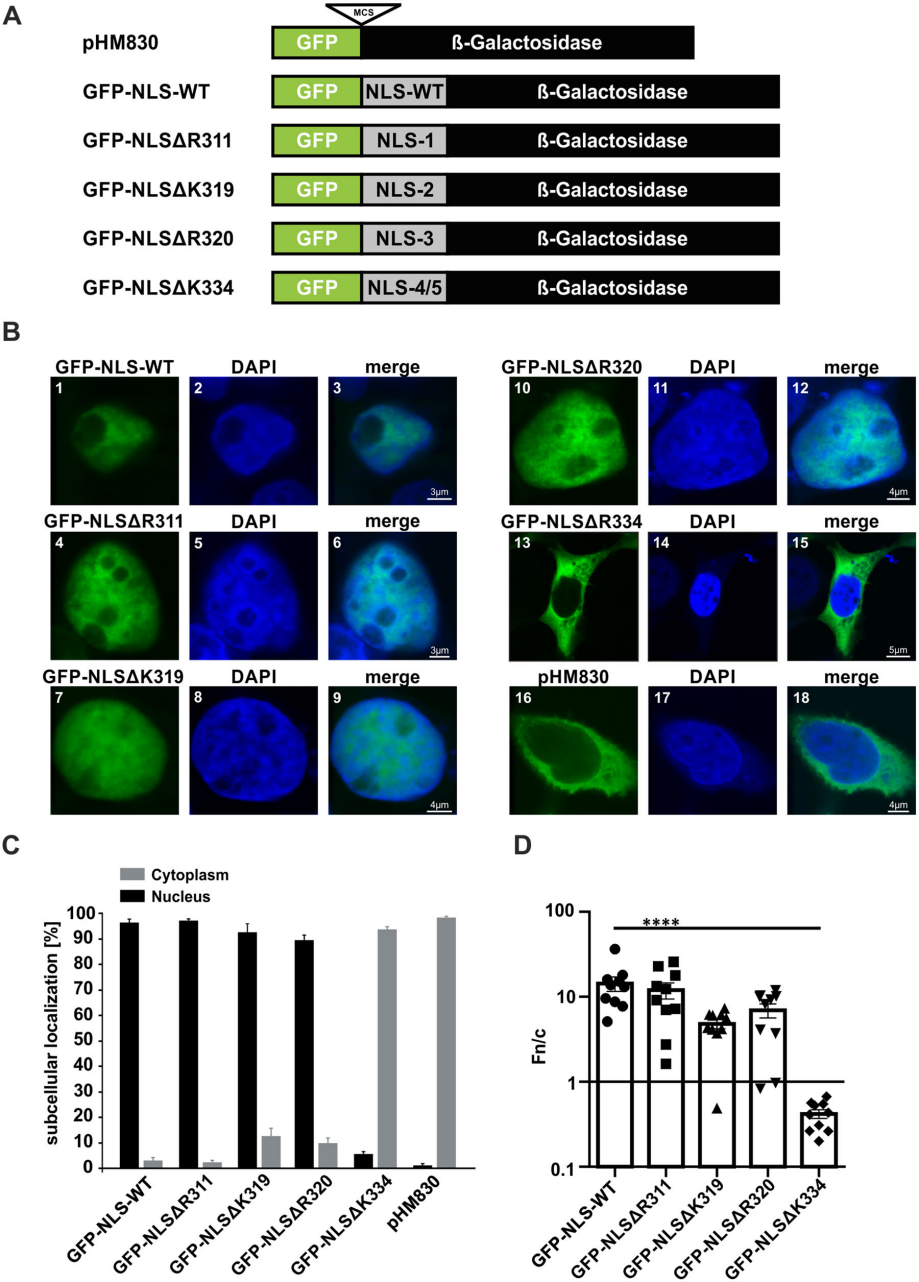
The ELL2 NLS relocalizes GFP‐β‐Gal fusion constructs to the nucleus. (A) Overview of an established NLS mapping system (pHM830) encoding an N‐terminal green fluorescent protein (GFP) and a C‐terminal beta‐galactosidase separated by a multiple cloning site (MCS). The MCS was utilized to introduce the NLS of ELL2‐WT (GFP‐NLS‐WT) and of the ELL2‐NLS deletion mutants as shown in Figure 3A (GFP‐NLS‐1 to GFP‐NLS‐4/5). (B) Immunofluorescence analysis was conducted in 293 T cells transfected with expression plasmids including GFP‐NLS‐WT‐β‐gal (GFP‐NLS‐WT, 1–3), GFP‐NLSΔR311‐β‐gal (GFP‐NLS‐1, 4–6), GFP‐NLSΔK319‐β‐gal (GFP‐NLS‐2, 7–9), GFP‐NLSΔR320‐β‐gal (GFP‐NLS‐3, 10–12), GFP‐NLSΔK334‐β‐gal (GFP‐NLS‐4/5, 13–15) or the empty vector pHM830 (GFP‐β‐gal, 16–18) without any NLS serving as control. After 48 h, cells were fixed with 2% para‐formaldehyde (1 h) followed by staining of the nuclei with DAPI. Images were acquired on a Leica TCS SP5 confocal laser scanning microscope with a 63 × 1.4 HCX PL APO CS oil immersion objective. Images of GFP expressed from pHM830 and the indicated ELL2‐NLS mutants (green), the nucleus (blue), and the merged stains are displayed. Scale bars indicate 3, 4, or 5 μm as indicated. (C) Quantitation of nuclear‐cytoplasmic localization of GFP‐NLS‐β‐Gal fusion constructs as displayed in B and Supporting Information S1: Figure S6. At least 382 cells in four optical fields of three independent experiments were analyzed. The frequency of cells expressing the respective GFP‐labeled constructs in the nucleus (black) or the cytoplasm (gray) are presented. (D) The ratio of mean fluorescence intensities between proteins located in the nucleus and the cytoplasm (*Fn/c*) in single cells like those shown in (B) expressing the indicated GFP‐NLS fusion constructs are shown. Each data point corresponds to quantification of a single cell, bars indicate mean values. Ten randomly selected cells per condition were analyzed, and values were compared to those obtained upon expression of ELL2‐WT using Kruskal–Wallis test and Dunn's multiple comparisons test (****p < 0.0001).

To assess whether the newly identified ELL2 NLS at aa 311–338 permits other proteins lacking a functional NLS sequence to enter the nucleus, we utilized an established NLS mapping system [40]. Briefly, this system includes an N‐terminal green fluorescent protein (GFP) serving as a fluorescent tag. GFP can penetrate the nucleus without an NLS sequence since its molecular weight is low (30 kDa). Therefore, the NLS mapping system also includes a C‐terminal β‐galactosidase (β‐gal), separated from the GFP‐expressing cassette by a multiple cloning site, thus, enabling the introduction of a putative NLS sequence into the system. Beyond, ß‐gal elevates the molecular weight to impede the passive diffusion of the GFP‐ß‐gal fusion protein into the nucleus [40, 41]. After introducing the isolated NLS of wildtype ELL2 and of ELL2‐NLS deletion mutants into the NLS mapping system by cloning (Figure 5A), the subcellular localization of GFP was analyzed upon transfection of 293 T cells (Figure 5B, Supporting Information S1: Figure S7) and quantitatively evaluated (Figure 5C). Western Blot analysis confirmed that all constructs are properly expressed (Supporting Information S1: Figure S8). The newly identified NLS of ELL2‐WT (aa 311–338) was found to re‐localize the NLS mapping system into the nucleus (Figure 5B, 1–3; Figure 5C), which was also confirmed by quantitative analysis reflecting nuclear accumulation of GFP‐NLS‐WT (*Fn/c* = 14.4 ± 2.8; Figure 5D) and indicating the functionality of the ELL2 NLS. Thus, the newly identified ELL2 NLS is capable of directing other proteins into the nucleus besides ELL2. When analyzing individual basic aa residues within the NLS, we could confirm that R311 is not crucial for the NLS sequence since its deletion in GFP‐NLS‐ΔR311 resulted in mainly nuclear localization of GFP (Figure 5B, 4–6; Figure 5B; Supporting Information S1: Figure S7B, 4–6) and high *Fn/c* values of 11.98 ± 2.6 (Figure 5D), consistent with previously observed results within full‐length ELL2 (Figure 4A, 4–6). Surprisingly, despite the predicted score of 0 (Figure 3A) and the predominantly cytoplasmatic localization when deleted in context of full‐length ELL2 (Figure 4A, 7–12; Supporting Information S1: Figure S5, 7–12; Figure 4B), both GFP‐NLSΔK319 and GFP‐NLS‐ΔR320 were mainly found in the nucleus (Figure 5B, 7–12; Figure 5C; Supporting Information S1: Figure S7B, 7–12). Quantitative evaluation of fluorescence intensities confirmed nuclear accumulation of both variants with *Fn/c* values of 4.8 ± 0.6 (GFP‐NLSΔK319) and 6.9 ± 1.3 (GFP‐NLS‐ΔR320), which were slightly but not significantly lower than those of GFP‐NLS‐WT (Figure 5D). This suggests that one basic aa at the adjacent positions 319 and 320 is sufficient to confer nuclear localization of the GFP‐based NLS mapping system (Figure 5), but not of full‐length ELL2 (Figure 4). However, the GFP‐NLS‐ΔK334 variant replicated previous findings pertaining to full‐length ELL2 (Figure 4A, 10–13; Figure 4B) and resulted in predominantly cytoplasmatic localization of GFP (Figure 5B, 13–15; 94% cytoplasmatic localization, Figure 5C) as confirmed by low *Fn/c* values of 0.4 ± 0.05 (Figure 5D). Finally, the NLS mapping system without any NLS sequence (pHM830) served as control and displayed almost exclusive localization in the cytoplasm (Figure 5B, 16–18; Figure 5C; Supporting Information S1: Figure S7B, 16–18), thus, confirming earlier findings [40, 41]. Summed up, the isolated NLS sequence of ELL2 has the ability to independently direct the transportation of other proteins into the nucleus without the involvement of the remaining ELL2 sequence, thus, validating our initial computational analysis.

## 4 Discussion

After the initial description of the transcription elongation factor ELL2 more than 25 years ago [1], ELL2 was found to play an important role in regulating transcription in various contexts including stress response, MYC‐driven cancer, secretory‐specific immunoglobulin heavy chain production, and viral transactivation of the human retroviruses HIV and HTLV‐1 [2, 3, 4, 7, 8, 14, 15, 18]. Although ELL2 is known to primarily localize in the nucleus [1, 4, 18, 42], an NLS for ELL2 has not been described yet. In this study, we identify the ELL2 NLS using computational analysis followed by imaging analysis of deletion mutants and by the use of an established NLS‐mapping system [40].

To date, several server‐based applications are available to detect putative NLS sequences. The cNLS Mapper software used in this study predicted a sequence stretch with an exceptional high score to serve as bipartite NLS. This prediction was based on homologies of the sequence stretch aa 311‐RDAVSSPQ**KR** *LLDSEFIDPLMN* **KK**A**R**IS‐338 to an (K/R)(K/R)*X*_10–12_(K/R)_3/5_ consensus sequence where two basic aa (bold) are separated by a linker (italics) from a second cluster of 3–5 basic aa (bold [27, 28, 29, 30]). Interestingly, the score predicted (score = 12) even exceeded the maximal scores given by cNLS Mapper for nuclear localization of a protein, which are between 8 and 10 [28], suggesting the presence of a very strong NLS in ELL2. Although the discovery of an NLS based on computational predictions proved to be rather accurate in the present study, this type of in silico analysis has also some limitations. NLS predictions usually result from a simplified model, which only takes into account the local aa composition, but does not consider the 3D structure of the intact protein [28]. Moreover, there is no strictly defined, generally valid NLS, and its aa composition can be rather variable [24, 27, 36, 43, 44]. Lastly, cNLS mapper was created based on *Saccharomyces cerevisiae* [28], which could lead to less accurate NLS identification in other species, thus, emphasizing the need for experimental validation of predictions.

Using ELL2 deletion mutants, we could indeed confirm that the sequence stretch being decisive for ELL2's nuclear localization is located within ELL2‐R1 (aa 7–353), which harbors the sequence of the novel NLS (aa 311–338). Interestingly, this conserved region R1 has been shown to be important for transcriptional elongation, RNA pol II binding, and for viral transactivation, respectively [1, 4, 16], suggesting that both localization and important biological functions of ELL2 are encoded on the same conserved domain. Moreover, the NLS sequences seems to be evolutionary conserved since we found highly homologous sequences in other species as well. Fine‐mapping of crucial basic aa being important for functionality of the novel ELL2 NLS by in silico deletions and bioinformatics predicted that basic aa K319 and R320, both upstream of the linker of the bipartite NLS, are more crucial for nuclear localization of ELL2 than basic aa K333/K334 directly succeeding the linker region. However, analysis of newly generated ELL2 deletion mutants in 293T cells showed, that both, aa pairs directly upstream and downstream of the linker, are essential for nuclear localization of ELL2. Interestingly, deletion of a more distantly located aa, R311, which is part of the predicted NLS, but not of the core NLS sequence, did not impair nuclear accumulation of ELL2 independent of in silico or in vitro analysis. In line with the computational analysis, the NLSΔR336 mutant displayed unclear localization, and quantitative analysis uncovered an almost evenly distribution of NLSΔR336 between the nucleus and the cytoplasm. Together, immunofluorescence analysis and the quantitative evaluation of fluorescence intensities in the nucleus and the cytoplasm demonstrated that the deletion of a single basic aa is sufficient to disrupt the functionality of the ELL2 NLS sequence. However, our data cannot discriminate between full‐length ELL2 and the alternatively translated ELL2 starting from Met186 since all proteins were analyzed via C‐terminal epitope tags. Surprisingly and against expectation, deletion of the novel NLS (aa 311–338) resulted in both nuclear and cytoplasmic localization of ELL2‐ΔNLS. A possible explanation for this unexpected behavior could be a change of ELL2 structure upon deletion of the NLS sequence stretch, which may expose one of the weaker NLS sequences that are predicted by *in silico* analysis (Figure 1; Supporting Information S1: Figure S1).

The NLS sequence (aa 311–338) was also analyzed individually and independent of the residual ELL2 sequence by using an established NLS mapping system consisting of GFP and β‐galactosidase which can also be analyzed by immunofluorescence and has been proven useful in other studies [40, 41, 45]. We decided against a fusion of NLS with GFP alone, because passive transport of GFP into the nucleus could already occur due to the low molecular weight [46]. The addition of a β‐galactosidase in the NLS mapping system increases the molecular weight to prevent this passive diffusion and offers an additional possibility for fluorescent staining [40]. The isolated ELL2 NLS sequence was sufficient to mediate transport of the mapping system into the nucleus, which was also confirmed by Fc/n analysis, thus, confirming the in silico predictions and the strength of the identified NLS. While analysis of GFP‐NLSΔR311 and GFP‐NLSΔK334 mutants recapitulated results from experiments analyzing full‐length ELL2 deletion mutants and showed strict cytoplasmatic localization, the behavior of GFP‐NLSΔK319 and GFP‐NLSΔR320 was different from experiments deleting K319 and R320 in the context of the whole ELL2 sequence: The discrepancy between the predominant nuclear localization of GFP‐NLS‐ΔK319/GFP‐NLS‐ΔR320 and the cytoplasmic localization of full‐length ELL2 upon introduction of these mutations underscores the necessity to verify findings obtained by computational analysis not only with the help of an NLS mapping system, but also within the full‐length sequence of the protein. Moreover, this suggests that one basic aa, either K or R, at this position is sufficient to mediate nuclear transport of the NLS mapping system, but not of wildtype ELL2. However, it is noteworthy that in silico predictions played a significant role in the identification of ELL2's NLS, and only the precise detection of aa being crucial for functionality of the NLS was enhanced by experimental analysis of ELL2‐NLS mutants. Together, our study could identify a functional and potent NLS in ELL2 and crucial basic aa being important for integrity of the NLS by combining in silico predictions with in vitro analysis of truncation mutants, deletion mutants, and an NLS mapping system. Yet, it remains to be determined whether the identified ELL2‐NLS interacts with importin‐α, thus initiating nuclear transport of ELL2 via the classical importin‐α/importin‐β pathway as typical for bipartite NLS sequences [24, 27, 33, 41]. Taken together, this work identified the NLS of ELL2 and highlights the importance of single basic aa within the NLS for nuclear localization of ELL2.

## Author Contributions

**Stephan Kohrt:** designed experiments, performed experiments, analyzed data, wrote manuscript. **Abarna Baheerathan:** performed experiments, analyzed data. **Jonas Prokscha:** performed experiments, analyzed data. **Alexandra Zwosta:** performed experiments, analyzed data. **Heinrich Sticht:** designed experiments, analyzed data, wrote manuscript, funding acquisition. **Andrea K. Thoma‐Kress:** designed experiments, analyzed data, wrote manuscript, funding acquisition, conceived of the study. All authors read and approved the final version of the manuscript.

## Conflicts of Interest

The authors declare no conflicts of interest.

## Supporting information

Supporting information.

## Data Availability

All data are contained within the manuscript or the supporting information. Additional data from imaging analysis will be shared upon request (AKTK; andrea.thoma-kress@uk-erlangen.de).
