# Supplementary material for: Identification of a Nuclear Localization Signal (NLS) in Human Transcription Elongation Factor ELL2
Source: Cell Biochem Funct. 2024 Nov 24;42(8):e70019. doi: 10.1002/cbf.70019 (PMC11586470; doi:10.1002/cbf.70019)
Supplement: Supplementary file 1 — Supporting information. [file CBF-42-e70019-s001.docx]

**Supplementary information**

**Identification of a nuclear localization signal (NLS) in human transcription elongation factor ELL2**

Stephan Kohrt^1^, Abarna Baheerathan^1^, Jonas Prokscha^1^, Alexandra Zwosta^1^, Heinrich Sticht^2,3^, Andrea K. Thoma-Kress^1,3*^

^1^ Institute of Clinical and Molecular Virology, Friedrich-Alexander-Universität Erlangen-Nürnberg (FAU), Erlangen, Germany

^2^ Division of Bioinformatics, Institute of Biochemistry, Friedrich-Alexander-Universität Erlangen-Nürnberg (FAU), Erlangen, Germany

^3^ FAU Profile Center Immunomedicine (FAU I-MED), Friedrich-Alexander-Universität Erlangen-Nürnberg (FAU), Schlossplatz 1, D-91054 Erlangen, Germany

^*^corresponding author: [andrea.thoma-kress@uk-erlangen.de](mailto:andrea.thoma-kress@uk-erlangen.de)

**Materials**

Materials included are described in the main article.

**Table S1. Oligonucleotides used for cloning.**

| **Primer** | **Sequence (5´ - 3´)** |
| --- | --- |
| ELL2-NLS1-fwd | ATCTCCTGTATGTTCTAGTGATGCTGTATCTTCTCCTC |
| ELL2-NLS1-rev | GAGGAGAAGATACAGCATCACTAGAACATACAGGAGAT |
| ELL2-NLS2-fwd | CTGTATCTTCTCCTCAGCGGCTTTTGGATTCAGA |
| ELL2-NLS2-rev | TCTGAATCCAAAAGCCGCTGAGGAGAAGATACAG |
| ELL2-NLS3-fwd | AATAAACTCTGAATCCAAAAGTTTCTGAGGAGAAGATACAGCA |
| ELL2-NLS3-rev | TGCTGTATCTTCTCCTCAGAAACTTTTGGATTCAGAGTTTATTG |
| ELL2-NLS5-fwd | TGAGATATTCGGGCTTTATTCATTAAAGGATCAATAAACTCTGAATCC |
| ELL2-NLS5-rev | GGATTCAGAGTTTATTGATCCTTTAATGAATAAAGCCCGAATATCTCA |
| GA-amp-fwd | TGAGGCACCTATCTCAGCGATCTG |
| GA-amp-rev | CAGATCGCTGAGATAGGTGCCTCA |
| GFP-NLS-WT-fwd | GAC**CTTAAG**AGAGATGCTGTATCTTCTCCTCAG**AAA**CGGCTTTTGGATTCA GAGTTTATTGATCCTTTAATGAATAAAAAAGCCCGAATATCTTCTAGAGAC |
| GFP-NLS-WT-rev | GTC**TCTAGA**AGATATTCGGGCTTTTTTATTCATTAAAGGATCAATAAACTCT GAATCCAAAAGCCGTTTCTGAGGAGAAGATACAGCATCTCTCTTAAGGTC |
| GFP-NLS1-fwd | GAC**CTTAAG**GATGCTGTATCTTCTCCTCAGAAACGGCTTTTGGATTCA GAGTTTATTGATCCTTTAATGAATAAA AAAGCCCGAATATCT TCTAGAGAC |
| GFP-NLS1-rev | GTC**TCTAGA**AGATATTCGGGCTTTTTTATTCATTAAAGGATCAATAAA CTCTGAATCCAAAAGCCGTTTCTGAGGAGAAGATACAGCATCCTTAAGGTC |
| GFP-NLS2-fwd | GAC**CTTAAG**AGAGATGCTGTATCTTCTCCTCAG**CGG**CTTTTGGATTCA GAGTTTATTGATCCTTTA ATGAATAAAAAAGCCCGAATATCTTCTAGAGAC |
| GFP-NLS2-rev | GTC**TCTAGA**AGATATTCGGGCTTTTTTATTCATTAA AGGATCAATAAACTCT GAATCCAAAAGCCGCTGAGGAGAAGATACAGCATCTCTCTTAAGGTC |
| GFP-NLS3-fwd | GAC**CTTAAG**AGAGATGCTGTATCTTCTCCTCAGAAACTTTTGGATTCAGA GTTTATTGATCCTTTAATGAATAAAAAAGCCCGAATATCTTCTAG AGAC |
| GFP-NLS3-rev | GTC**TCTAGA**AGATATTCGGGCTTTTTTATTCATTAAAGGATCAATAAACTC TGAATCCAAAAGTTTCTGAGGAGAAGATACAGCATCTCTCTTAA GGTC |
| GFP-NLS4/5-fwd | GAC**CTTAAG**AGAGATGCTGTATCTTCTCCTCAGAAACGGCTTTTGGATTC AGAGTTTATTGATCCTTTAATGAATAAAGCCCGAATATCTTCTAGAGAC |
| GFP-NLS4/5-rev | GTC**TCTAGA**AGATATTCGGGCTTTATTCATTAAAGGATCAATAAACTC TGAATCCAAAAGCCGTTTCTGAGGAGAAGATACAGCATCTCTCTTAA GGTC |

**
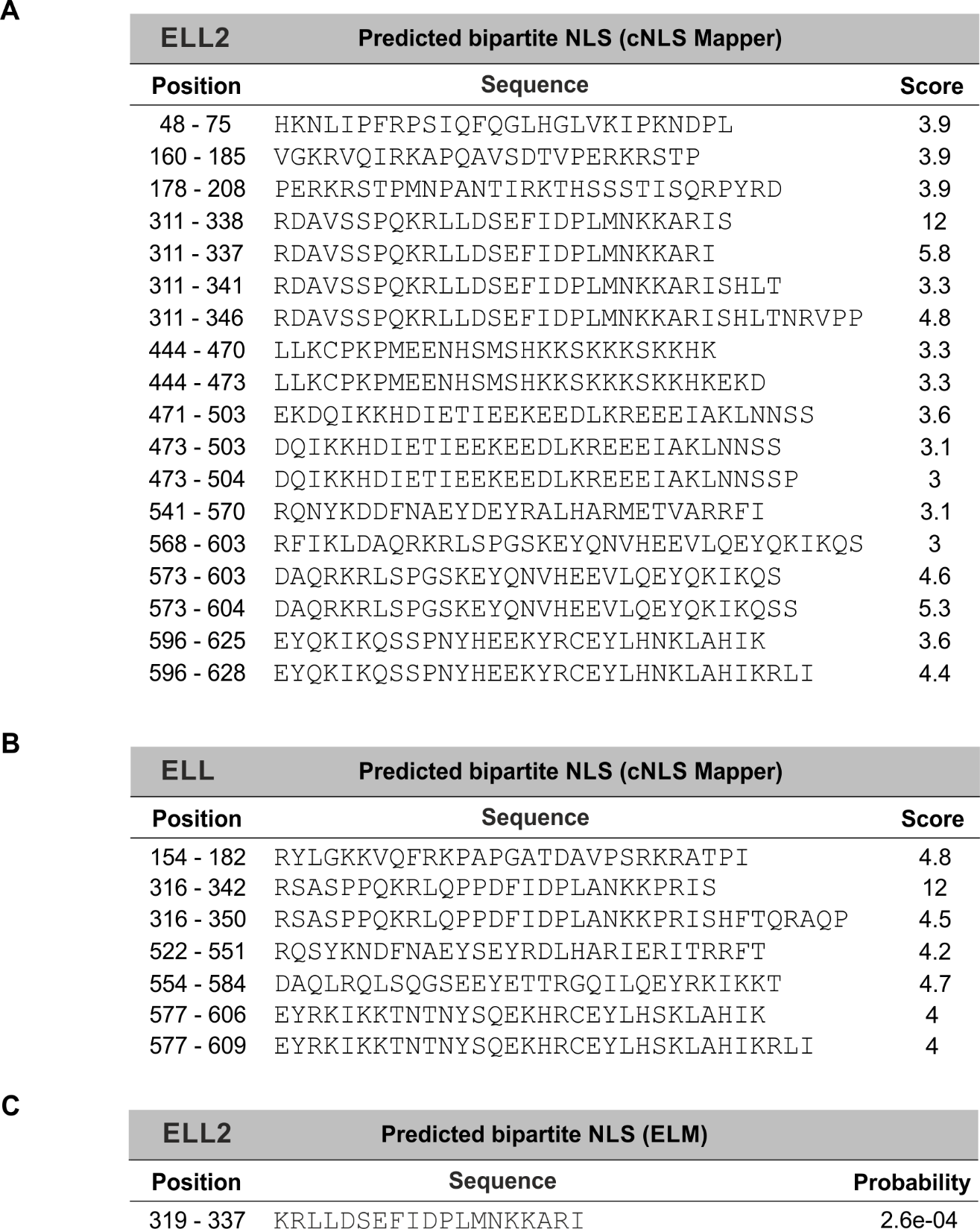
**

**Figure S1. Prediction of a bipartite nuclear localization signal (NLS) in ELL2 using computational analysis.** Amino acid (aa) sequences of the bipartite NLS sequences in **(A)** ELL2 and **(B)** ELL as predicted by cNLS Mapper and in **(C)** ELL2 as predicted by ELM. Positions of the respective sequences within full-length **(A, C)** ELL2 or **(B)** ELL, respectively, and **(A, B)** the scores or **(C)** probability of the respective predictions are indicated.


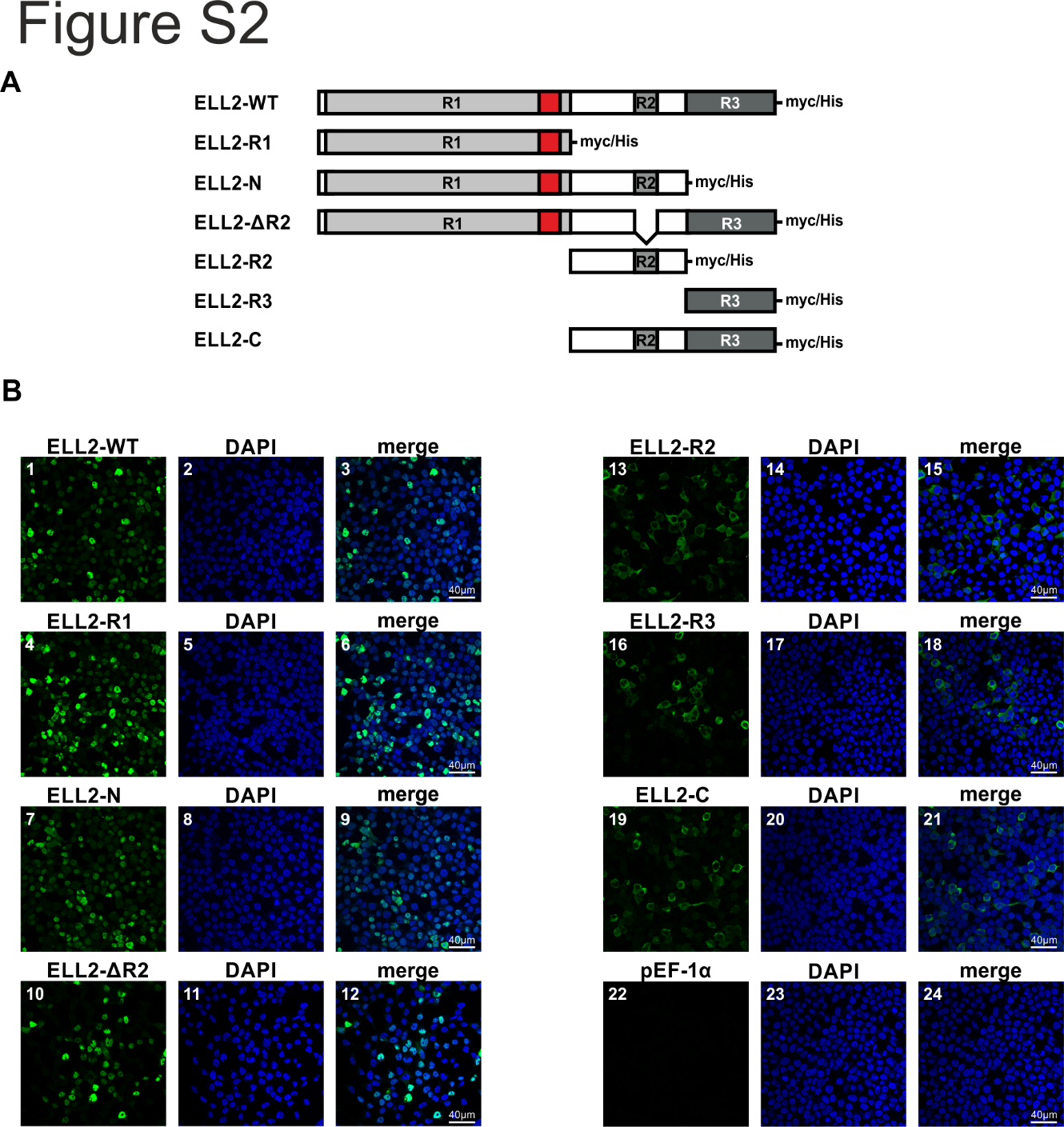


**Figure S2. The R1 region is crucial for nuclear localization of ELL2. (A)** Schematic representation of ELL2 wildtype (ELL2-WT) and ELL2 truncation mutants with C-terminal myc-his tag. R1, R2, R3, conserved regions 1,2, 3, respectively. Red box: localization of putative NLS. **(B)** Immunofluorescence analysis of ELL2-WT, ELL2 truncations, and the nucleus was conducted in 293T cells. Cells were transfected with 1 µg of expression plasmids pEF-ELL2-myc (ELL2-WT, 1-3), pEF-ELL2-R1-myc (ELL2-R1, 4-6), pEF-ELL2-N-myc (ELL2-N, 7-9), pEF-ELL2-ΔR2-myc (ELL2-ΔR2, 10-12), pEF-ELL2-R2-myc (ELL2-R2, 13-14), pEF-ELL2-R3-myc (ELL2-R3, 16-18), pEF-ELL2-C-myc (ELL2-C, 19-21) or an empty vector control (pEF1α, 22-24). After 48 h, cells were stained with primary mouse anti-myc followed by anti-mouse Alexa Fluor 488® (green) antibodies. Nuclei were counterstained with DAPI (blue). Images were acquired on a Leica TCS SP5 confocal laser scanning microscope with a 63×1.4 HCX PL APO CS oil immersion objective. Representative overview images corresponding to Figure 2 and depicting ELL2-WT and ELL2 truncations (green), the nucleus (blue), and the merged stains are displayed. Scale bars indicate 40 µm.

**
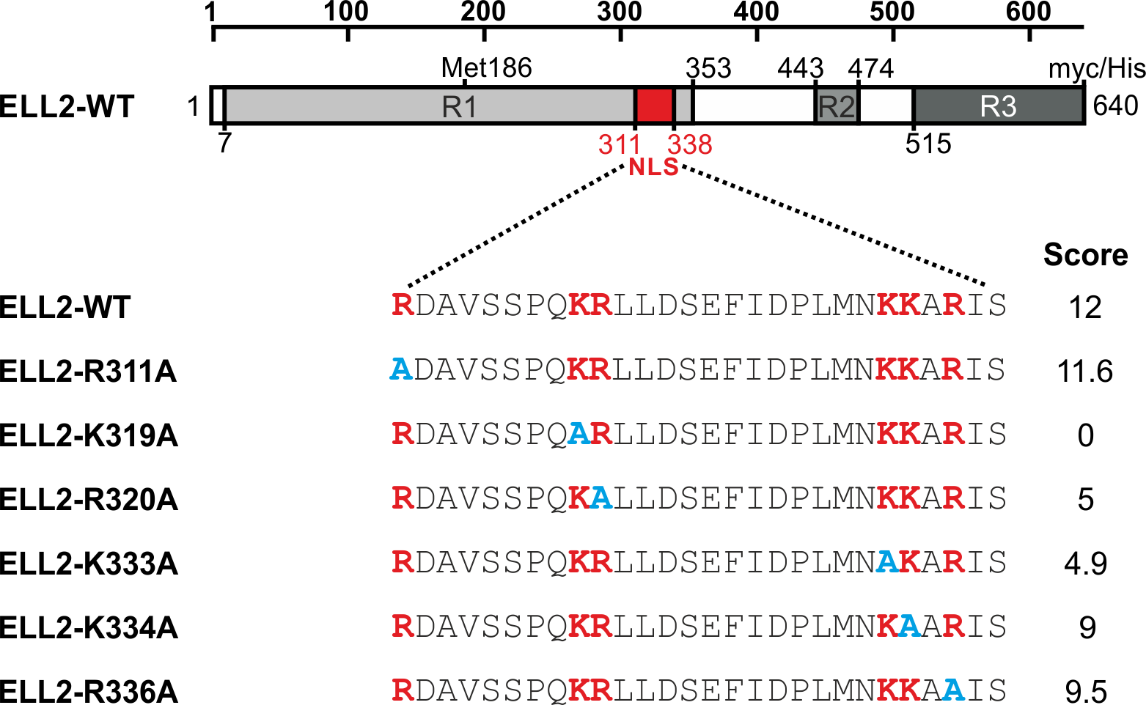
**

**Figure S3.** Schematic overview of ELL2-WT and *in silico* analysis of ELL2 mutants. The ELL2-WT aa sequence carrying the predicted nuclear localization signal (NLS, aa 311-338), *in silico* designed ELL2 mutants with alanine substitutions (A, blue) of all basic amino acids (bold, red) within the predicted ELL2 NLS were analyzed by cNLS Mapper. Scores of the respective sequences are indicated.

**
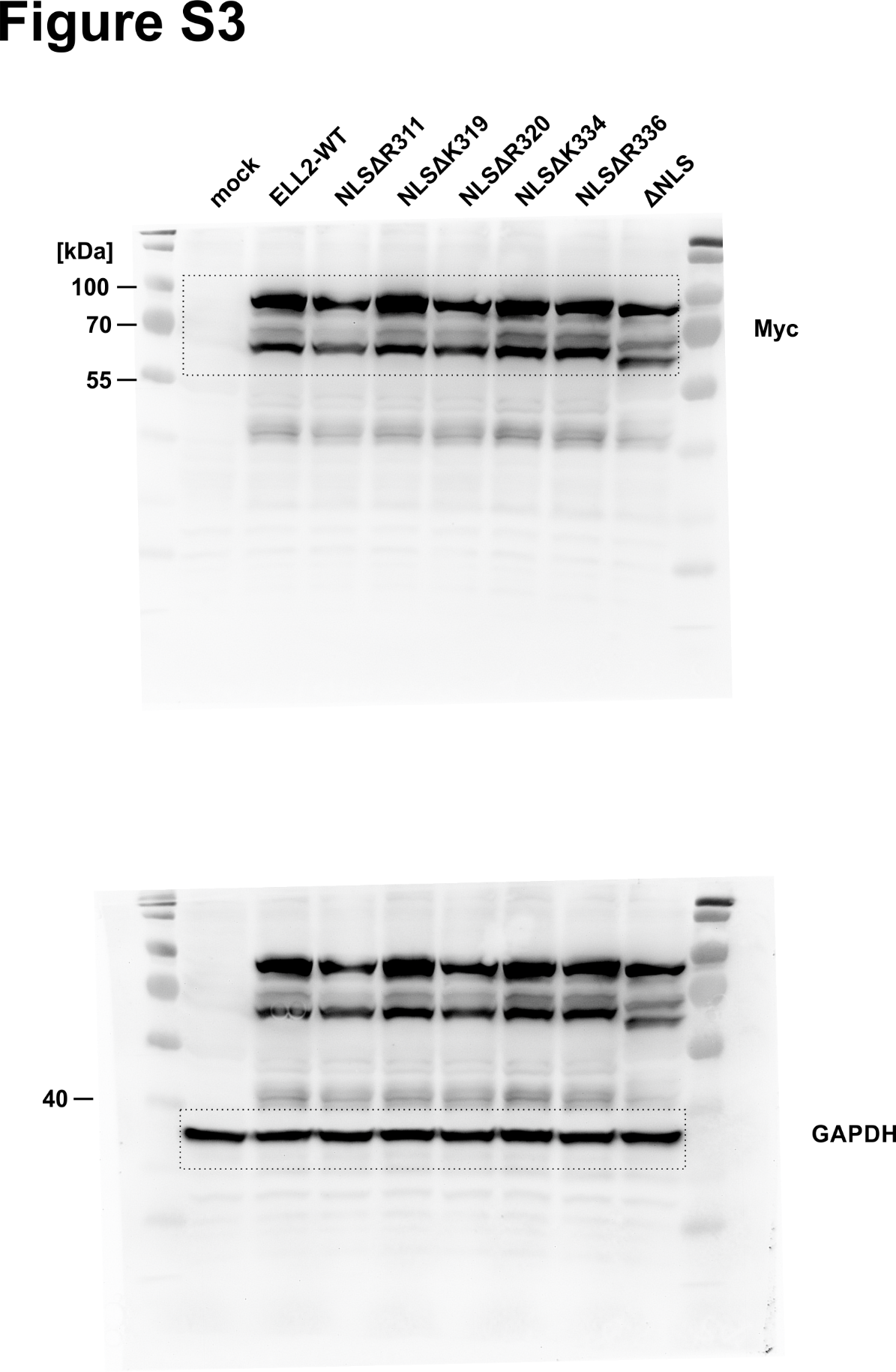
**

**Figure S4. In silico and in vitro mutagenesis of the ELL2 NLS.** Test expression of ELL2-NLS deletion mutants upon transfection of 293T cells with 1 µg of myc-tagged expression plasmids, including pEF-1α-ELL2-myc (ELL2), ELL2 truncations pEF-1α-ELL2-NLS-1-myc (NLSΔR311), pEF-1α-ELL2-NLS-2-myc (NL2ΔK319), pEF-1α-ELL2-NLS-3-myc (NLSΔR320), pEF-1α-ELL2-NLS-5-myc (NLSΔK334), and the control vector pEF-1α (mock). After 48 h, Western Blot analysis was performed using antibodies specific for myc and the housekeeping gene glyceraldehyde 3-phosphate dehydrogenase (GAPDH). Uncropped image corresponding to Figure 3B, elements used are highlighted with a dotted line.


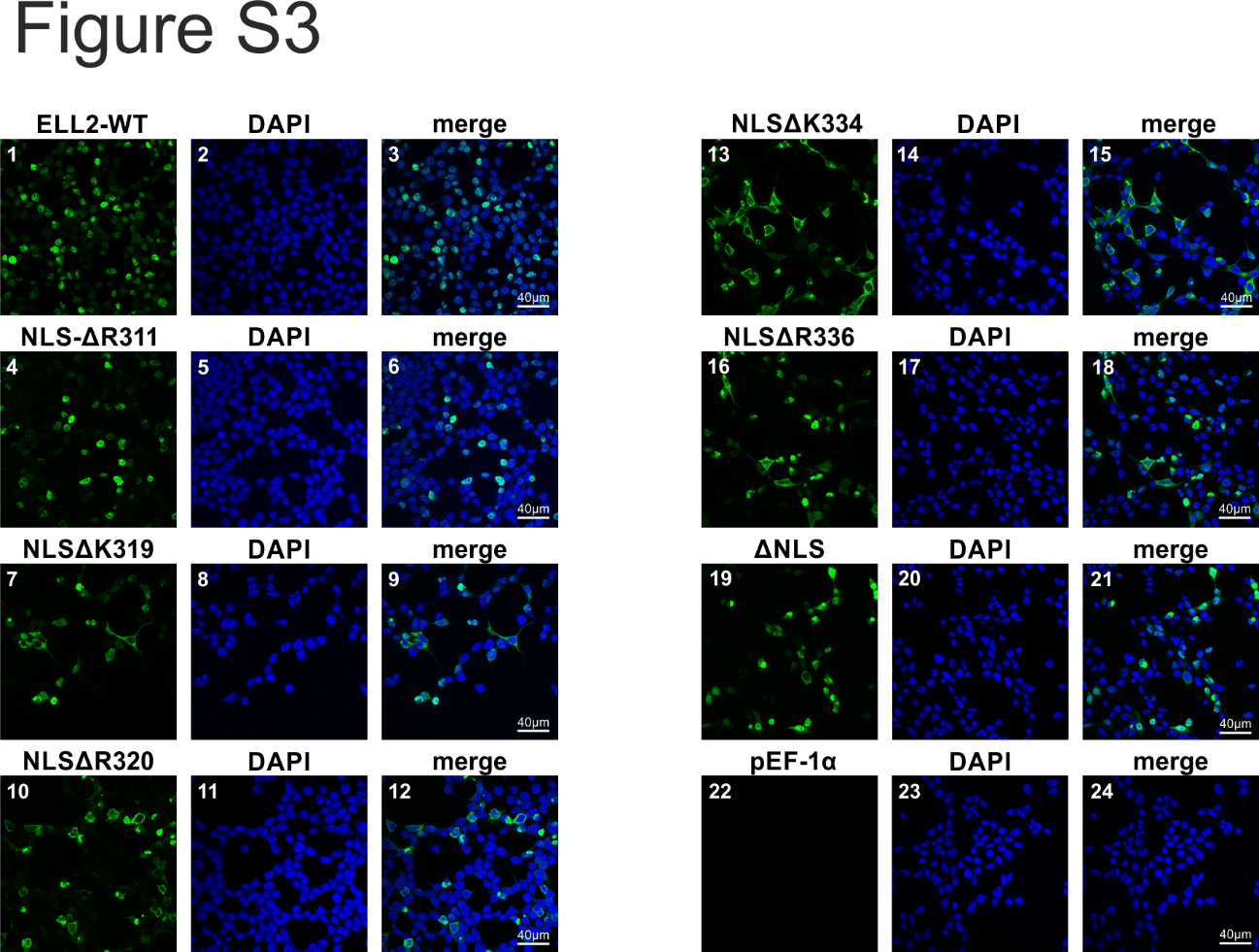


**Figure S5. Basic residues K319, R320, K333/K334 are essential for the nuclear localization of ELL2.** Immunofluorescence analysis of ELL2-WT, ELL2-NLS mutants and the nucleus in 293T cells transfected with the expression plasmids (1 µg each) pEF-1α-ELL2-myc (ELL2-WT, 1-3), ELL2 deletion mutants pEF-1α-ELL2-NLS-1-myc (NLSΔR311, 4-6), pEF-1α-ELL2-NLS-2-myc (NL2ΔK319, 7-9), pEF-1α-ELL2-NLS-3-myc (NLSΔR320, 10-12), pEF-1α-ELL2-NLS-4/5-myc (NLSΔK334, 13-15), pEF-1α-ELL2-NLS-6-myc (NLSΔR336, 16-21), pEF-1α-ELL2-∆NLS-myc (∆NLS, 22-27), or the empty vector pEF-1α (28-30) as negative control. After 48 h, cells were stained with primary mouse anti-myc followed by anti-mouse Alexa Fluor 488® (green) antibodies. Nuclei were counterstained with DAPI (blue). Images were acquired on a Leica TCS SP5 confocal laser scanning microscope with a 63×1.4 HCX PL APO CS oil immersion objective. Representative overview images corresponding to Figure 4 and depicting ELL2-WT and ELL2-NLS deletion mutants (green), the nucleus (blue), and the merged stains are displayed. Scale bars indicate 40 µm as indicated.


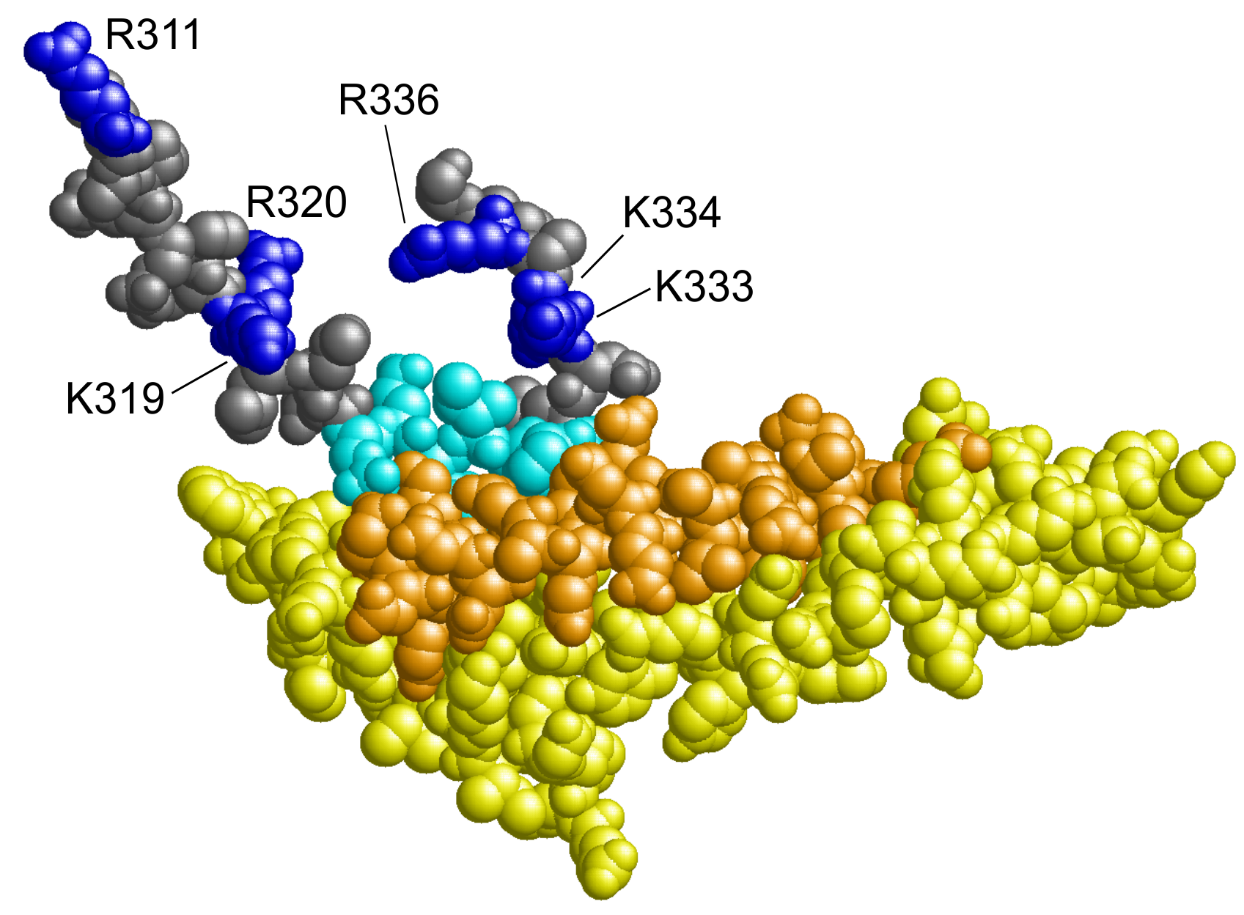


**Figure S6. Deletion of the primary NLS might cause the exposure of a secondary weak NLS in ELL2.** Model of ELL2 indicating the interaction between the primary NLS region (aa 311-338) and the R3 domain (yellow/orange). The primary NLS region is colored in grey, and the R3-interacting stretch (aa 324-330) is highlighted in cyan. This stretch corresponds to the central spacer of the bipartite NLS. The basic residues of the NLS are shown in blue and are labelled. A second weak bipartite NLS region in R3 (residues 541-570) is colored in orange. This weak NLS is partially masked by the interaction with residues 324-330 (cyan) in full-length ELL2, but might become functional after deletion of residues 311-338 in ELL2-ΔNLS.

**
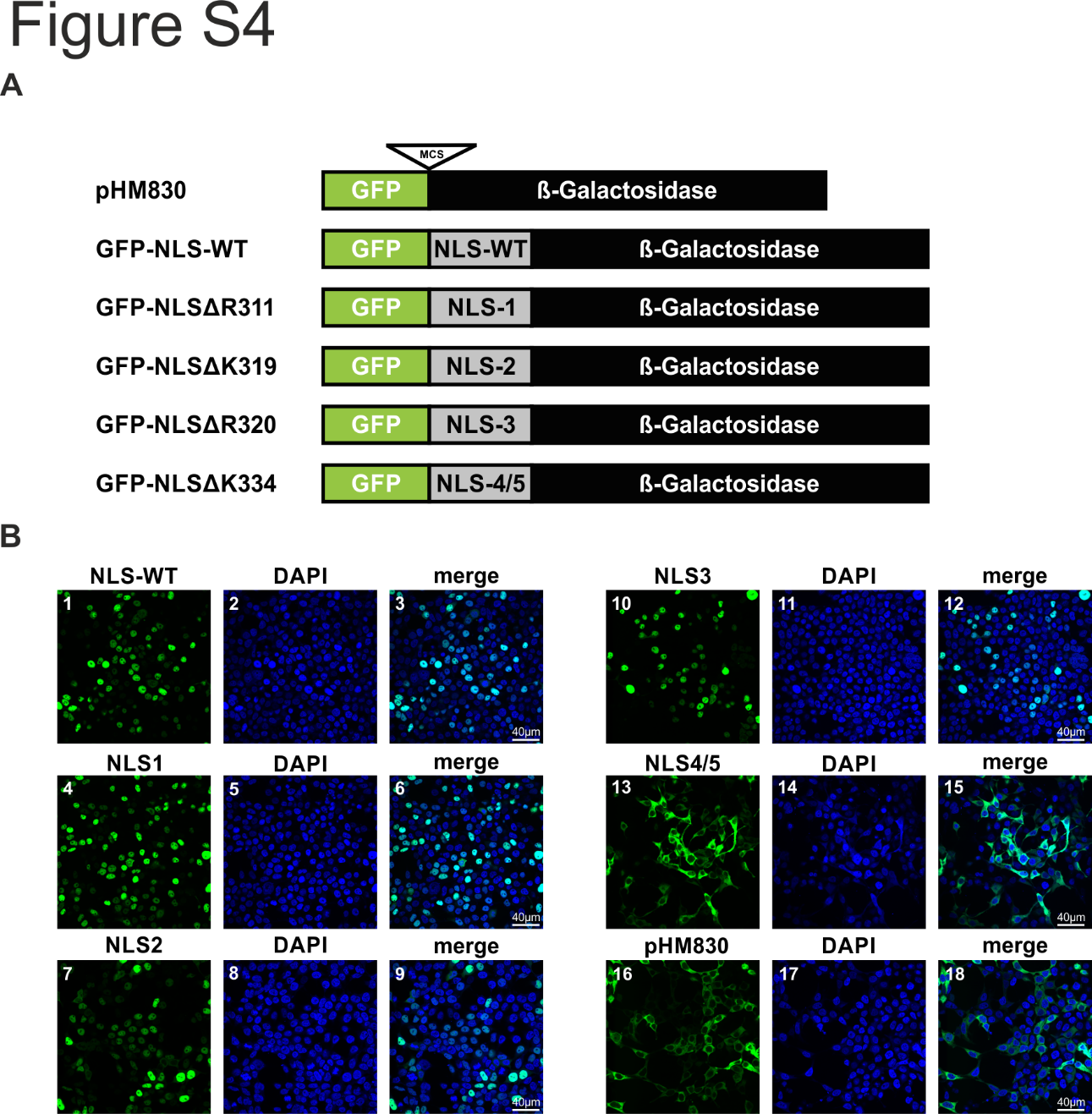
**

**Figure S7. The ELL2 NLS relocalizes GFP-β-Gal fusion proteins to the nucleus. (A)** Overview of an established NLS mapping system (pHM830) encoding an N-terminal green fluorescent protein (GFP) and a C-terminal beta-galactosidase separated by a multiple cloning site (MCS). The MCS was utilized to introduce the NLS of ELL2-WT (GFP-NLS-WT) and of the ELL2-NLS deletion mutants as shown in Figure 3A (GFP-NLS-1 to GFP-NLS-4/5). **(B)** Immunofluorescence analysis was conducted in 293T cells transfected with expression plasmids including GFP-NLS-WT-β-gal (GFP-NLS-WT, 1-3), GFP- NLSΔR311-β-gal (GFP-NLS-1, 4-6), GFP-NLSΔK319-β-gal (GFP-NLS-2, 7-9), GFP-NLSΔR320-β-gal (GFP-NLS-3, 10-12), GFP-NLSΔK334-β-gal (GFP-NLS-4/5, 13-15) or the empty vector pHM830 (GFP-β-gal, 16-18) without any NLS serving as control. After 48 h, cells were fixed with 2% para-formaldehyde (1 h) followed by staining of the nuclei with DAPI. Images were acquired on a Leica TCS SP5 confocal laser scanning microscope with a 63×1.4 HCX PL APO CS oil immersion objective. Representative overview images corresponding to Figure 5 and showing GFP expressed from pHM830 and the indicated ELL2-NLS mutants (green), the nucleus (blue), and the merged stains. Scale bars indicate 40 µm.

**
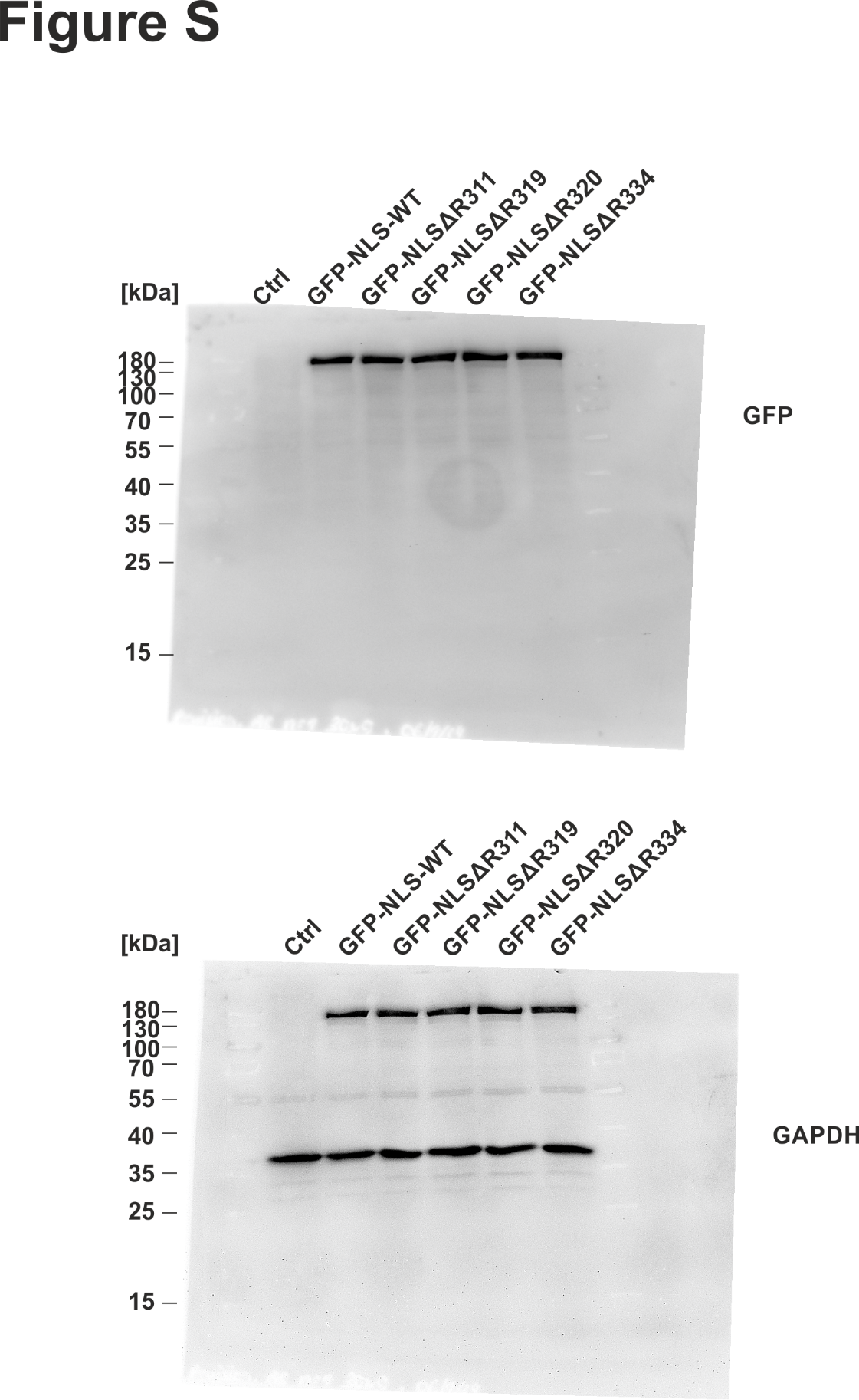
**

**Figure S8. GFP-NLS-β-Gal fusion proteins are properly expressed.** Expression of the indicated GFP-NLS-β-Gal fusion proteins including GFP-NLS-WT-β-gal (GFP-NLS-WT), GFP-NLSΔR311-β-gal (GFP-NLS-1), GFP-NLSΔK319-β-gal (GFP-NLS-2), GFP-NLSΔR320-β-gal (GFP-NLS-3), GFP-NLSΔK334-β-gal (GFP-NLS-4/5) compared to cells expressing no GFP (ctrl.) was analyzed by Western Blot at 48 h post transfection of 293T cells. Antibodies specific for GFP and the housekeeping gene glyceraldehyde 3-phosphate dehydrogenase (GAPDH) were used and uncropped images are depicted.
